# Supplementary material for: Multidimensional scaling informed by F-statistic: Visualizing grouped microbiome data with inference
Source: PLoS Comput Biol. 2026 Apr 1;22(4):e1014102. doi: 10.1371/journal.pcbi.1014102 (PMC13108906; doi:10.1371/journal.pcbi.1014102)
Supplement: S1 File — (PDF) [file pcbi.1014102.s001.pdf]

Supporting Information of "Multidimensional scaling informed by F-statistic: Visualizing grouped microbiome data with inference"

Hyungseok Kim[1†], Soobin Kim[2†], Jeffrey A. Kimbrel[3], Megan M. Morris[3], Xavier Mayali[3], and Cullen R. Buie[1*]

[1]Massachusetts Institute of Technology, Cambridge, MA, USA
[2]University of California, Davis, Davis, CA, USA
[3]Lawrence Livermore National Laboratory, Livermore, CA, USA
[†]These authors contributed equally to this work.
[*]Correspondence: crb@mit.edu.

List of Supplementary Information

Appendix A. F-MDS objective function and mapping between F-ratios 2
Appendix B. Majorization-Minimization algorithm 5
Appendix C. Hyperparameter selection procedure 7
Appendix D. Human gut microbiome dataset 8
Appendix E. Neural network model and architecture 9
Appendix F. Computational complexity 10

Appendix A. F-MDS objective function and mapping between F-ratios

In this section, we derive an objective function to compute F-informed MDS. We define the objective function $O_{\text{FMDS}}(\mathbf{Z})$ based on the MDS stress function by adding a confirmatory term that facilitates PERMANOVA p-values to be similar when the function is minimized. First, a local regression function, $f_{\mathbf{z}}(F_{\mathbf{x}})$ is designed to minimize the difference between p-values testing group differences under the original S- and two-dimensional embedding through the confirmatory term (see Eq 5 of the main text). These p-values are obtained from an empirical distribution of the pseudo-F statistics generated by label permutations [1] (Eq 4, main text). In specific, for a given set of label groups $[y_i]_{i=1}^N$ with G groups, the pseudo F-statistics are expressed as

$$\begin{aligned} F_{\mathbf{x}}^{\Pi} &= \left(\frac{\sum_{i,j} d_{ij}^2}{G \sum_{i,j} d_{ij}^2 \, \mathbb{I}\{y_i^{\Pi} = y_j^{\Pi}\}} - 1 \right) \cdot \frac{N-G}{G-1}, \\ F_{\mathbf{z}}^{\Pi} &= \left(\frac{\sum_{i,j} \|\mathbf{z}_i - \mathbf{z}_j\|_2^2}{G \sum_{i,j} \|\mathbf{z}_i - \mathbf{z}_j\|_2^2 \, \mathbb{I}\{y_i^{\Pi} = y_j^{\Pi}\}} - 1 \right) \cdot \frac{N-G}{G-1}, \end{aligned} \tag{S1}$$

where the superscript Π denotes permutation. It should be noted that the pseudo-F ratios are distributed differently depending on the dimensionality (e.g., original space vs. two-dimensional embedding). To evaluate and compare statistical significance across dimensions, we introduce a mapping function $f_{\mathbf{z}} : F_{\mathbf{x}} \to F_{\mathbf{z}}$ defined from pairs of ratios $(F_{\mathbf{x}}^{\Pi}, F_{\mathbf{z}}^{\Pi})$. Each element is obtained by sorting repeated F-ratios generated from randomly permuted label sets, i.e., $\mathbf{y}^{\Pi_k}$ for $k = 1, \cdots, K$. The figure below illustrates how the permuted F-ratios in each dimension can differ in scale. Sorting the ratios by magnitude enables identification of the pair $(F_{\mathbf{x}}, F_{\mathbf{z}})$ from unpermuted labels with respect to the reference curve using the mapping function $f_{\mathbf{z}}$.

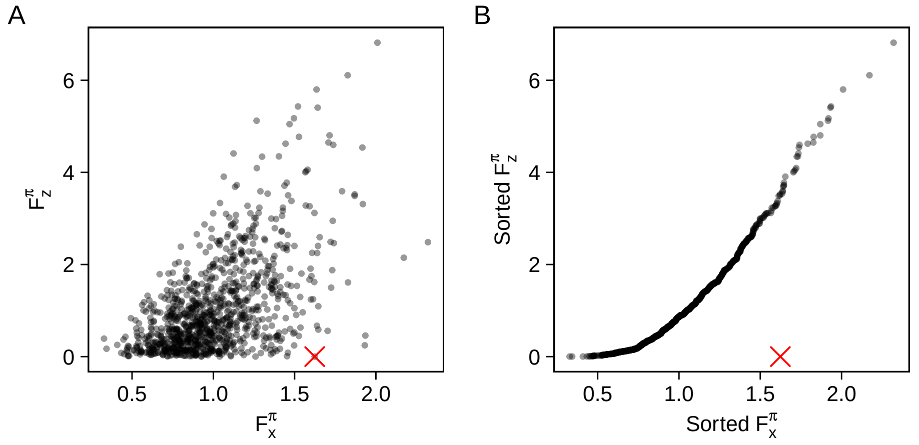

Fig A. Mapping of pseudo-F statistics between two dimensionalities. The F-ratios were computed using a binary semisynthetic dataset generated with SparseDOSSA [10], with labels sets permuted 1,000 times. The ratios from each dimension were (A) paired according to the same label permutation and (B) sorted by magnitude to perform local regression. The F-ratios from the unpermuted label set are indicated by red cross marks.

Using the mapping function $f_{\mathbf{z}}$, we derive an exact expression for the confirmatory term introduced in the F-MDS objective function. The proposed form $|F_{\mathbf{z}} - f_{\mathbf{z}}(F_{\mathbf{x}})|$ measures how ac-

curately the 2D representation reflects the S-dimensional distance structure regarding the sample labels. A difference close to zero indicates that no further updates to the representation are required (denoted $\mathbf{Z}^*$). Substituting the confirmatory term $|F_\mathbf{z} - f_\mathbf{z}(F_\mathbf{x})|$ with Equation S1, we define $\epsilon_{ij} := \mathbb{1}\{y_i^\Pi = y_j^\Pi\}$ and write

$$\mathbf{Z}^* = \arg\min_{\mathbf{Z}} |F_\mathbf{z} - f_\mathbf{z}(F_\mathbf{x})| \tag{S2}$$

$$= \arg\min_{\mathbf{Z}} \left| \frac{N-G}{G-1} \cdot \left(\frac{\sum_{i,j} \|\mathbf{z}_i - \mathbf{z}_j\|_2^2}{G \sum_{i,j} \epsilon_{ij} \|\mathbf{z}_i - \mathbf{z}_j\|_2^2} - 1 \right) - f_\mathbf{z}(F_\mathbf{x}) \right| \tag{S3}$$

$$= \arg\min_{\mathbf{Z}} \left| \frac{\sum_{i,j} \|\mathbf{z}_i - \mathbf{z}_j\|_2^2}{G \sum_{i,j} \epsilon_{ij} \|\mathbf{z}_i - \mathbf{z}_j\|_2^2} - 1 - \frac{G-1}{N-G} \cdot f_\mathbf{z}(F_\mathbf{x}) \right| \tag{S4}$$

$$\approx \arg\min_{\mathbf{Z}} \left| \sum_{i,j} \|\mathbf{z}_i - \mathbf{z}_j\|_2^2 - G \sum_{i,j} \epsilon_{ij} \|\mathbf{z}_i - \mathbf{z}_j\|_2^2 \cdot \left(1 + \frac{G-1}{N-G} \cdot f_\mathbf{z}(F_\mathbf{x}) \right) \right| \tag{S5}$$

$$= \arg\min_{\mathbf{Z}} \left| \sum_{i,j} \left[1 - G\epsilon_{ij} \left(1 + \frac{G-1}{N-G} \cdot f_\mathbf{z}(F_\mathbf{x}) \right) \right] \|\mathbf{z}_i - \mathbf{z}_j\|_2^2 \right|, \tag{S6}$$

where Equation S5 follows from the numerator of Equation S4.

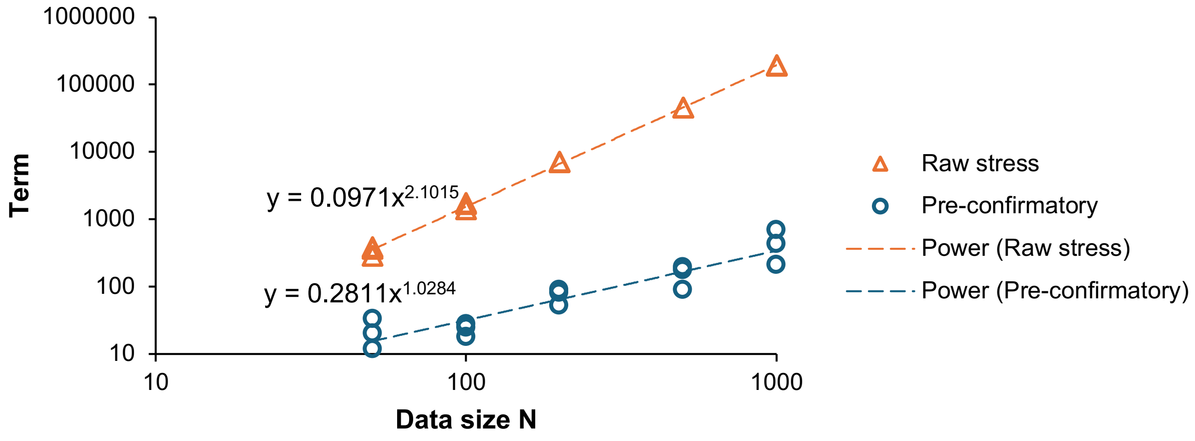

Fig B. Scaling analysis of raw stress and pre-confirmatory terms across data size $N \in \{50, 100, 200, 500, 1000\}$ with semisynthetic data.

Finally, the exact F-MDS confirmatory term is derived through a scaling analysis of expression S6 by comparing its value to that of the raw stress (main text Eq 1). Binary semisynthetic datasets generated by SparseDOSSA [10], with sizes N varying from 50 to 1000, were used to compute the following quantities,

$$\text{Raw stress} = \sum_{i,j} (d_{ij} - \|\mathbf{z}_i - \mathbf{z}_j\|_2)^2 \tag{S7}$$

$$\text{Pre-confirmatory} = \sum_{i,j} \left[1 - G\epsilon_{ij} \left(1 + \frac{G-1}{N-G} \cdot f_\mathbf{z}(F_\mathbf{x}) \right) \right] \|\mathbf{z}_i - \mathbf{z}_j\|_2^2. \tag{S8}$$

Computing these two values across different data sizes N, as shown in the figure above, indicates that the raw stress and pre-confirmatory terms scale differentially with N, with approximate powers 2.1 and 1.0, respectively. While the theoretical foundations remain to be established for such zero-inflated, log-normally distributed data, empirical analysis suggests that the size effect can be removed by multiplying the pre-confirmatory term with N.

By combining raw stress and confirmatory terms with hyperparameter λ, we conclude the derivation of the F-MDS objective function as

$$O_{\mathrm{FMDS}}(\mathbf{Z}) = \sum_{i,j}(d_{ij} - \|\mathbf{z}_i - \mathbf{z}_j\|_2)^2 + \lambda N \left| \sum_{i,j} \left[1 - G\epsilon_{ij} \left(1 + \frac{G-1}{N-G} \cdot f_{\mathbf{z}}(F_{\mathbf{x}}) \right) \right] \|\mathbf{z}_i - \mathbf{z}_j\|_2^2 \right|. \quad \text{(S9)}$$

Appendix B. Majorization-Minimization algorithm

We seek a configuration $\mathbf{Z}^* = (\mathbf{z}_1^*, \cdots \mathbf{z}_N^*) \in \mathbb{R}^{N\times 2}$ that minimizes the F-MDS objective function, $O_{\text{FMDS}}(\mathbf{Z})$. Previous work by Witten & Tibshirani [22] suggests that MDS-based ordinations can be computed by applying the Majorization (or Majorize-Minimization) algorithm to a quadratic expression in $\mathbf{Z}$. Although our F-MDS objective function is also quadratic in $\mathbf{Z}$ and the Majorization algorithm is applicable, the coefficient of the confirmatory term depends on the sample size N and the hyperparameter λ, as shown in Equation S9. For the algorithm to consistently produce the optimized $\mathbf{Z}$, it is important to define an update rule that is independent of the sample size. Therefore, we excluded the coefficient N from the confirmatory term of Equation S9. Additionally, the convexity of the majorized form was ensured by constraining the range of λ to [0,1]:

$$\text{minimize } O^*(\mathbf{Z}) = \sum_{i,j}(d_{ij} - \|\mathbf{z}_i - \mathbf{z}_j\|_2)^2 + \lambda \left| \sum_{i,j} \left[1 - G\epsilon_{ij}\left(1 + \frac{G-1}{N-G} \cdot f_{\mathbf{z}}(F_{\mathbf{x}})\right)\right] \|\mathbf{z}_i - \mathbf{z}_j\|_2^2 \right|.$$

In detail, the algorithm sequentially seeks a point $\mathbf{z}_k^*$ for every $k = 1, \cdots N$, while other configuration points remain fixed. This is written as

$$\mathbf{z}_k^* = \arg\min_{\mathbf{z}_k} O^*(\mathbf{Z}|\mathbf{z}_1, \cdots \mathbf{z}_{k-1}, \mathbf{z}_{k+1}, \mathbf{z}_N) \tag{S10}$$

$$= \arg\min_{\mathbf{z}_k} \sum_{j=1}^{N}(d_{jk} - \|\mathbf{z}_j - \mathbf{z}_k\|_2)^2 + \lambda\delta(\mathbf{z}) \sum_{j=1}^{N} \left[1 - G\epsilon_{jk}\left(1 + \frac{G-1}{N-G} f_{\mathbf{z}}(F)\right)\right] \|\mathbf{z}_j - \mathbf{z}_k\|_2^2 \tag{S11}$$

$$= \arg\min_{\mathbf{z}_k} \sum_{j=1}^{N} \left[1 + \lambda\delta(\mathbf{z})\left(1 - G\epsilon_{jk}\left(1 + \frac{G-1}{N-G} f_{\mathbf{z}}(F)\right)\right)\right] \|\mathbf{z}_k - \mathbf{z}_j\|_2^2 - 2d_{jk}\|\mathbf{z}_k - \mathbf{z}_j\|_2 \tag{S12}$$

where we define $\delta(\mathbf{z}) \coloneqq \text{sign}\left\{ \sum_{i,j} \left[1 - G\epsilon_{ij}\left(1 + \frac{G-1}{N-G} f_{\mathbf{z}}(F)\right)\right] \|\mathbf{z}_i - \mathbf{z}_j\|_2^2 \right\}$.

As described by [2], applying the algorithm starts with majorizing with Equation S12,

$$\sum_{j=1}^{N} \left[1 + \lambda\delta(\mathbf{z})\left(1 - G\epsilon_{jk}\left(1 + \frac{G-1}{N-G} f_{\mathbf{z}}(F)\right)\right)\right] \|\mathbf{z}_k - \mathbf{z}_j\|_2^2 - 2d_{jk} \frac{\sum_{s=1}^{2}(z_{ks} - z_{js})(\tilde{z}_{ks} - z_{js})}{\|\tilde{\mathbf{z}}_k - \mathbf{z}_j\|_2}, \tag{S13}$$

where $\tilde{\mathbf{z}}_k$ remain fixed while updating $\mathbf{z}_k$. We further assume that a change of mapping function $f_{\mathbf{z}}(F)$ is negligible and that $\delta(\mathbf{z})$ remains constant during the iteration (e.g., a small change in $\mathbf{z}_k$ from metric MDS). These allow us to approximate Equation S13 with a quadratic expression in terms of $\mathbf{z}$ and proceed to its minimization, given its convexity. To find the minimum at $\mathbf{z}_k = \mathbf{z}_k^\dagger$, a derivative is taken with respect to z_{ks} and is set to zero. Applying it to Equation S13, we have

$$0 = \sum_{j=1}^{N} \left[1 + \lambda\delta(\mathbf{z})\left(1 - G\epsilon_{jk}\left(1 + \frac{G-1}{N-G} f_{\mathbf{z}}(F)\right)\right)\right] (z_{ks}^\dagger - z_{js}) - d_{jk} \frac{\tilde{z}_{ks} - z_{js}}{\|\tilde{\mathbf{z}}_k - \mathbf{z}_j\|_2}, \forall s = 1, 2 \tag{S14}$$

and for a balanced design where $\sum_{j=1}^{N} \epsilon_{jk} = N/G$ with every $k = 1, \cdots N$,

$$\left(N - \frac{(G-1)N\lambda\delta(\mathbf{z}) f_{\mathbf{z}}(F)}{N-G}\right) z_{ks}^\dagger = \sum_{j=1}^{N} \left[1 + \lambda\delta(\mathbf{z})\left(1 - G\epsilon_{jk}\left(1 + \frac{G-1}{N-G} f_{\mathbf{z}}(F)\right)\right)\right] z_{js} + d_{jk} \frac{\tilde{z}_{ks} - z_{js}}{\|\tilde{\mathbf{z}}_k - \mathbf{z}_j\|_2}. \tag{S15}$$

Rewriting Equation S15 in a vector form, we finally obtain the update rule of $\mathbf{Z}$ as

$$\begin{aligned}\mathbf{z}_k \leftarrow & \frac{N-G}{N(N-G)-(G-1)N\lambda\delta(\mathbf{z})f_{\mathbf{z}}(F)} \times \\ & \left\{ \sum_{j=1}^{N} \left[1+\lambda\delta(\mathbf{z})\left(1-G\epsilon_{jk}\left(1+\frac{G-1}{N-G}f_{\mathbf{z}}(F)\right)\right)\right]\mathbf{z}_j + d_{jk}\frac{\mathbf{z}_k-\mathbf{z}_j}{\|\mathbf{z}_k-\mathbf{z}_j\|_2} \right\}.\end{aligned} \tag{S16}$$

Finally, a stopping rule is defined for the majorization-minimization algorithm based on the computed PERMANOVA p-values. Noting that the p-value in the representation $\mathbf{Z}$ (denoted $p_{\mathbf{z}}$) initially decreases then begins to oscillate between 0 and 1, the rule is set to trigger at the last epoch before the updated difference $|p_{\mathbf{z}} - p_{\mathbf{x}}|$ exceeds its value at the previous epoch.

Appendix C. Hyperparameter selection procedure

We adopt a quantitative framework to select the optimal hyperparameter λ for computing F-MDS. First, we introduce an objective function $f_{\mathrm{obj}}(\lambda)$ to quantify the relationship between λ and algorithmic performance. Earlier analysis of F-MDS indicates that higher values of λ increase its convergence rate, measured by the number of epochs $n_{\mathrm{epoch}}(\lambda)$, but increase the deviation from the original distance structure (measured, for example, by Pearson correlation ρ_{dist} between pairwise distances). To balance these effects without bias, we applied min-max normalization to the metrics and defined

$$f_{\mathrm{obj}}(\lambda) = n_{\mathrm{epoch}}(\lambda)\big|_{\mathrm{norm}} \cdot (1 - \rho_{\mathrm{dist}}(\lambda))\big|_{\mathrm{norm}}, \tag{S17}$$

with each subscript $(\cdot|_{\mathrm{norm}})$ denoting that the values were rescaled between 0 and 1 across all training data. The grid search was performed for $\lambda \in [0.15, 1]$; for λ less than 0.15, for example, $p_{\mathbf{z}}$ did not converge to $p_{\mathbf{z}}$ until fifty computational epochs for at least one dataset. For λ greater than unity, on the other hand, algorithm did not converge (or it was not examined) because the majorized objective function (Equation S13) was no longer convex and caused the solution to diverge.

Computations of $f_{\mathrm{obj}}(\lambda)$ show that the objective function is largely minimized at a moderate to high λ value (see **??**A). The optimal hyperparameter, denoted as λ_{min}, was calculated as 0.725$\pm$0.186 (mean$\pm$sd), with all optimal values except one greater than or equal to 0.5 (**??**B). We found no statistically significant relationship between λ_{min} and data size N ($p = 0.686$, Spearman correlation test). This further suggests the hyperparameter selection procedure using grid search is robust against overfitting.

Note that F-MDS produces a 2D representation uniquely determined by the input data matrix, which is unlike standard machine learning studies. As this simple approach still avoids the risk of overfitting, it suffices for hyperparameter selection.

Appendix D. Human gut microbiome dataset

We retrieved two sets of human gut microbiome data from a publicly available repository [12], previously generated from Shotgun Metagenomic Sequencing, where the reads were merged at the genus level [17]. The first dataset contained a gut microbiome derived from 118 healthy and 114 liver cirrhosis patients from a single study [16]. The second includes samples from a human gut of 217 healthy and 223 patients with type 2 diabetes (T2D) from two separate studies [6, 15].

For both datasets, a phylogenetic tree was generated using phyloT [8] where branch lengths were uniformly assigned with unity, resulting in 268 (cirrhosis) and 216 (T2D) features or taxa respectively. Taxonomy and abundance table were obtained using the phylogenetic tree and the merged reads, respectively, which were then integrated via `phyloseq` (Bioconductor v3.18). Pairwise distance matrix $\mathbf{D}$ was computed based on the weighted Unifrac [9].

Appendix E. Neural network model and architecture

We sought to compare our F-MDS with neural network models that are used for dimensionality reduction. To convert compositional microbial abundance into a matrix with its phylogenetic information, we implemented PopPhy-CNN [17] architecture. Each converted matrix reflected a phylogenetic tree structure by bacterial 16S rRNA amplicon (amplicon sequence variant or ASV) and its relative abundance which is normalized by cumulative sum scaling (CSS) [13]. Thirty-six bacterial community samples were retrieved and re-analyzed from the previous work [7]. The samples represent balanced design of diatom-associated community with and without presence of the host. Each compositional sample was converted to a 2D array sized 10×42. The data was randomly split into training and validation sets (6 and 30 each) using the stratified K-Fold.

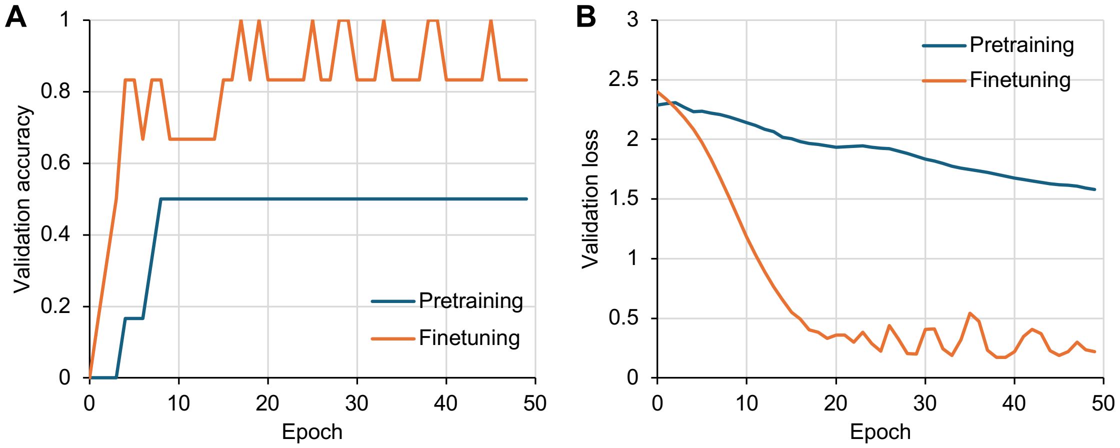

Fig A. Performance of self-supervised learning classifier by 50 training epochs. (A) Validation accuracy and (B) validation loss measured by categorical cross-entropy using bacterial community data represented as an image using PopPhy-CNN.

The neural network architecture included an encoder consisting of one Gaussian noise filter, two 2D convolution layers (with kernel size of 5 by 3), and one fully connected layer with 32 output nodes. A self-supervised learning framework using SimCLR [4] was chosen to explore its capability of arranging the microbiome data. The data augmentation was performed by applying random brightness and contrast filters with following parameters: (0.6, 0.2) for pretraining, (0.3, 0.1) for finetuning. In a pretraining step of SimCLR, a model was constructed by compiling encoder, projection head (two dense layers each of 32 output nodes), and one dense layer (10 output nodes). Site 1 and 2 datasets were individually trained and evaluated (30, 6 samples each) for the pretraining step, resulting a linear probing accuracy of 53.3% after 50 training epochs (see Figure above). In the following finetuning step, the encoder is added with linear probe, resulting in a validation accuracy higher than 83.3% after 50 epochs (see figure below). The trained encoder was used to obtain a 32 nodes-sized feature for each microbial community sample. The 32-dimensional feature was used for evaluating this neural network model with quality metrics. Pairwise distance was calculated using L_2-squared metric to obtain Stress-1 and Shepard plot.

Appendix F. Computational complexity

We provide an upper bound of computational complexity of majorization algorithm for performing F-MDS. Like most iteration-based optimizations, the computation cost of majorization was estimated on the basis of a single step. We discuss the time complexity of each step outlined in Algorithms 1 and 2 of the main text.

For computing the mapping function (Algorithm 1), a pseudo-F-ratio is computed from a set of permuted labels y^{Π} and each of input matrices d, $\mathbf{z}$. Each computation takes $\mathcal{O}(N^2)$ operations with N being the sample size. The step repeats for a number of iteration, e.g., $p = 999$, resulting in $\mathcal{O}(2pN^2)$ operations. Additional steps are taken to sort the lists of permuted F-ratios with $\mathcal{O}(2N \log N)$. In total, the complexity is $\mathcal{O}(2pN^2 + 2N \log N)$.

For majorization step (Algorithm 2), the F-ratio is computed once and is mapped to $f_{\mathbf{z}}(F)$, taking $\mathcal{O}(N^2 + \log N)$ operations. Next, the sign of F-MDS confirmatory term $\delta(\mathbf{Z})$ is obtained and the step takes $\mathcal{O}(N^2)$ operations. Finally, the 2D representation $\mathbf{Z}$ is updated for every point, taking $\mathcal{O}(N^2)$ operations. Therefore, the complexity for one iteration of the majorization algorithm is $\mathcal{O}(3N^2 + \log N)$.

In summary, the computational cost of performing F-MDS (unit iteration) is $\mathcal{O}(2pN^2 + 3N^2 + 2N \log N + \log N) \approx \mathcal{O}(pN^2)$. It is compared with other dimension reduction methods which is summarized below.

Table A. Comparison of time complexity between different dimensionality reduction methods.

Method	Complexity	Algorithm
F-informed MDS[1]	$\mathcal{O}(pN^2)$	Majorization [3]
MDS	$\mathcal{O}(N^3)$	Eigendecomposition [19]
	$\mathcal{O}(N \log N)$	Divide-and-conquer [23]
Supervised MDS[1]	$\mathcal{O}(N^2)$	Majorization [22]
UMAP	$\mathcal{O}(N^{1.14})$	NN-descent [5, 11]
t-SNE[1]	$\mathcal{O}(N^2)$	Gradient descent [21]
	$\mathcal{O}(N \log N)$	Barnes-Hut or dual-tree [20]
Isomap	$\mathcal{O}(N^2 \log N)$	Dijkstra's [14, 18]

[1]Corresponds to a single iteration and does not represent a total complexity.

References

[1] Anderson, M. J. A new method for non-parametric multivariate analysis of variance. *Austral Ecology 26*, 1 (2001), 32–46.

[2] Borg, I., and Groenen, P. *Confirmatory MDS*. Springer New York, New York, NY, 1997, pp. 181–197.

[3] Borg, I., and Groenen, P. *A Majorization Algorithm for Solving MDS*. Springer New York, New York, NY, 1997, pp. 169–197.

[4] Chen, T., Kornblith, S., Norouzi, M., and Hinton, G. A simple framework for contrastive learning of visual representations. In *Proceedings of the 37th International Conference on Machine Learning* (2020), vol. 119, Proceedings of Machine Learning Research, pp. 1597–1607.

[5] Dong, W., Moses, C., and Li, K. Efficient k-nearest neighbor graph construction for generic similarity measures. In *Proceedings of the 20th International Conference on World Wide Web* (New York, NY, USA, 2011), WWW '11, Association for Computing Machinery, p. 577–586.

[6] Karlsson, F. H., Tremaroli, V., Nookaew, I., Bergström, G., Behre, C. J., Fagerberg, B., Nielsen, J., and Bäckhed, F. Gut metagenome in european women with normal, impaired and diabetic glucose control. *Nature 498*, 7452 (2013), 99–103.

[7] Kim, H., Kimbrel, J. A., Vaiana, C. A., Wollard, J. R., Mayali, X., and Buie, C. R. Bacterial response to spatial gradients of algal-derived nutrients in a porous microplate. *The ISME Journal 16*, 4 (2022), 1036–1045.

[8] Letunic, I. phylot: a tree generator, 12/8/2023 2023.

[9] Lozupone, C. A., Hamady, M., Kelley, S. T., and Knight, R. Quantitative and qualitative beta diversity measures lead to different insights into factors that structure microbial communities. *Applied and Environmental Microbiology 73*, 5 (2007), 1576–85.

[10] Ma, S., Ren, B., Mallick, H., Moon, Y. S., Schwager, E., Maharjan, S., Tickle, T. L., Lu, Y., Carmody, R. N., Franzosa, E. A., Janson, L., and Huttenhower, C. A statistical model for describing and simulating microbial community profiles. *PLOS Computational Biology 17*, 9 (2021), e1008913.

[11] McInnes, L., Healy, J., and Melville, J. Umap: Uniform manifold approximation and projection for dimension reduction. *arXiv* (2018), arXiv:1802.03426.

[12] Pasolli, E., Truong, D. T., Malik, F., Waldron, L., and Segata, N. Machine learning meta-analysis of large metagenomic datasets: Tools and biological insights. *PLoS Computational Biology 12*, 7 (2016), e1004977.

[13] Paulson, J. N., Stine, O. C., Bravo, H. C., and Pop, M. Differential abundance analysis for microbial marker-gene surveys. *Nature Methods 10* (2013).

[14] Pedregosa, F., Varoquaux, G., Gramfort, A., Michel, V., Thirion, B., Grisel, O., Blondel, M., Prettenhofer, P., Weiss, R., Dubourg, V., Vanderplas, J., Passos, A., Cournapeau, D., Brucher, M., Perrot, M., and Duchesnay, E. Scikit-learn: Machine learning in python. *Journal of Machine Learning Research 12* (2011), 2825–2830.

[15] Qin, J., Li, Y., Cai, Z., Li, S., Zhu, J., Zhang, F., Liang, S., Zhang, W., Guan, Y., Shen, D., Peng, Y., Zhang, D., Jie, Z., Wu, W., Qin, Y., Xue, W., Li, J., Han, L., Lu, D., Wu, P., Dai, Y., Sun, X., Li, Z., Tang, A., Zhong, S., Li, X., Chen, W., Xu, R., Wang, M., Feng, Q., Gong, M., Yu, J., Zhang, Y., Zhang, M., Hansen, T., Sanchez, G., Raes, J., Falony, G., Okuda, S., Almeida, M., LeChatelier, E., Renault, P., Pons, N., Batto, J.-M., Zhang, Z., Chen, H., Yang, R., Zheng, W., Li, S., Yang, H., Wang, J., Ehrlich, S. D., Nielsen, R., Pedersen, O., Kristiansen, K., and Wang, J. A metagenome-wide association study of gut microbiota in type 2 diabetes. *Nature 490*, 7418 (2012), 55–60.

[16] Qin, N., Yang, F., Li, A., Prifti, E., Chen, Y., Shao, L., Guo, J., Le Chatelier, E., Yao, J., Wu, L., Zhou, J., Ni, S., Liu, L., Pons, N., Batto, J. M., Kennedy, S. P., Leonard, P., Yuan, C., Ding, W., Chen, Y., Hu, X., Zheng, B., Qian, G., Xu, W., Ehrlich, S. D., Zheng, S., and Li, L. Alterations of the human gut microbiome in liver cirrhosis. *Nature 513*, 7516 (2014), 59–64.

[17] Reiman, D., Metwally, A. A., Sun, J., and Dai, Y. PopPhy-CNN: A phylogenetic tree embedded architecture for convolutional neural networks to predict host phenotype from metagenomic data. *IEEE Journal of Biomedical and Health Informatics 24*, 10 (2020), 2993–3001.

[18] Tenenbaum, J. B., de Silva, V., and Langford, J. C. A global geometric framework for nonlinear dimensionality reduction. *Science 290*, 5500 (2000), 2319–23.

[19] Torgerson, W. S. Multidimensional scaling: I. theory and method. *Psychometrika 17*, 4 (1952), 401–419.

[20] Van Der Maaten, L. Accelerating t-sne using tree-based algorithms. *Journal of Machine Learning Research 15*, 1 (2014), 3221–3245.

[21] van der Maaten, L., and Hinton, G. Visualizing data using t-SNE. *Journal of Machine Learning Research 9*, 86 (2008), 2579–2605.

[22] Witten, D. M., and Tibshirani, R. Supervised multidimensional scaling for visualization, classification, and bipartite ranking. *Computational Statistics & Data Analysis 55*, 1 (2011), 789–801.

[23] Yang, T., Liu, J., McMillan, L., and Wang, W. A fast approximation to multidimensional scaling. In *Proceedings of the ECCV Workshop on Computation Intensive Methods for Computer Vision (CIMCV)* (2006).
