# Supplementary figures and images for: Multidimensional scaling informed by F-statistic: Visualizing grouped microbiome data with inference
Source: PLoS Comput Biol. 2026 Apr 1;22(4):e1014102. doi: 10.1371/journal.pcbi.1014102 (PMC13108906; doi:10.1371/journal.pcbi.1014102)

**A**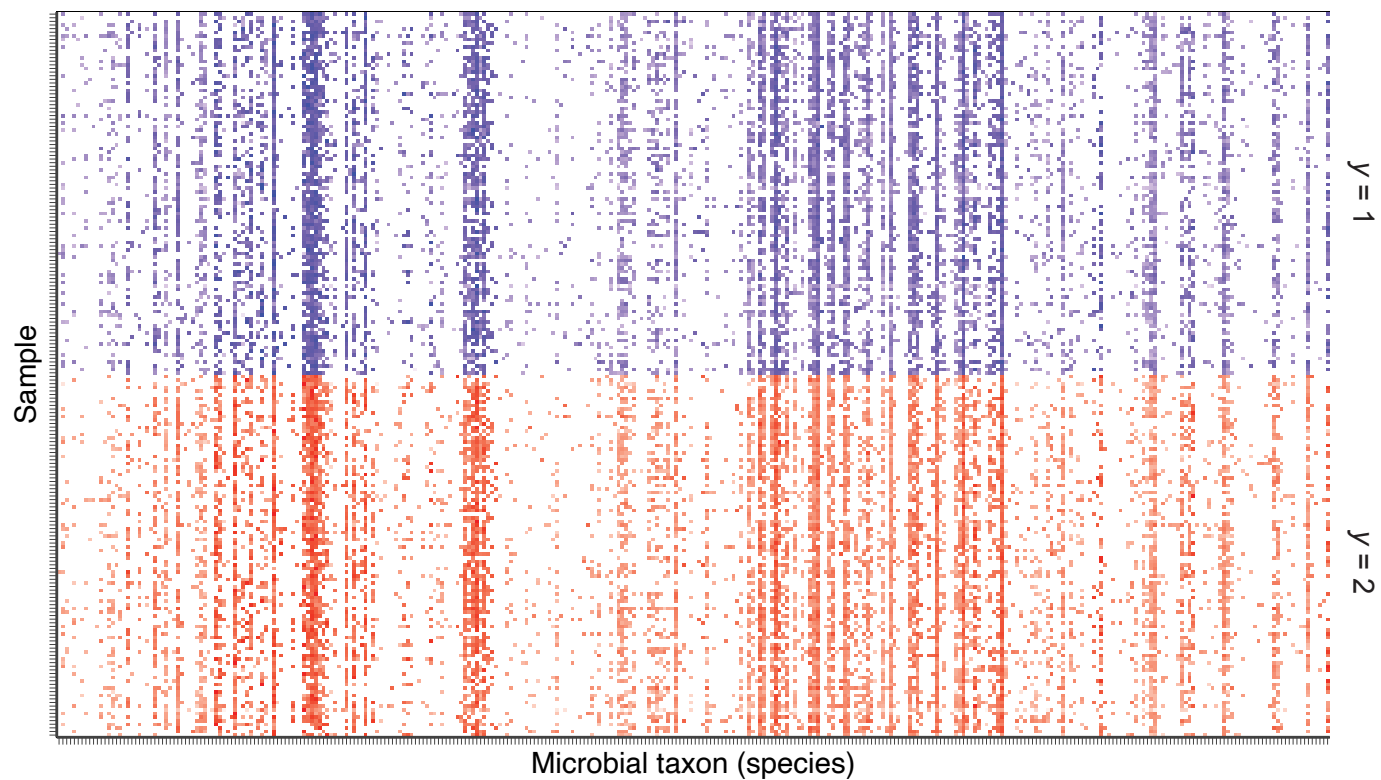**B**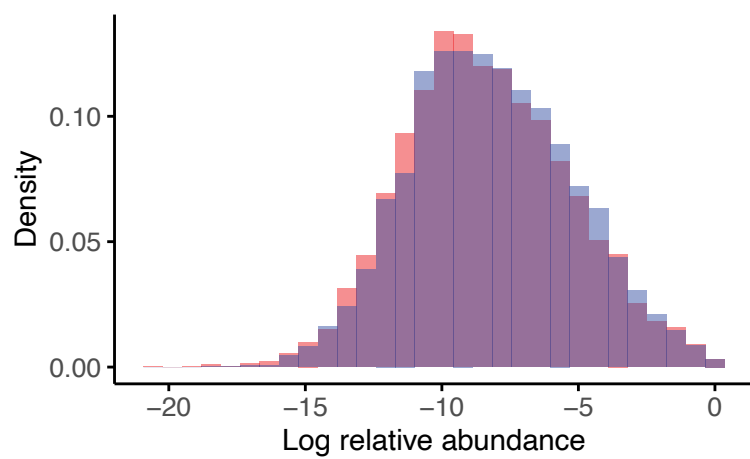

Group 1 2

**C**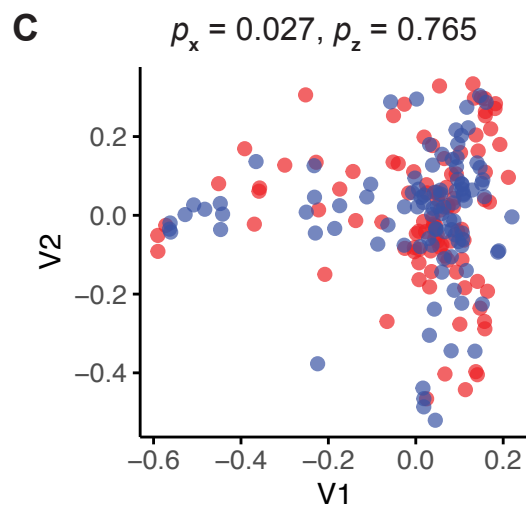

Group 1 2

Supplement: S1 Fig — (A) Heatmap of its log-scaled relative abundance by its microbial taxa and sample number. (B) Density histogram of the data and (C) principal coordinates analysis (PCoA) and PERMANOVA p-values based on PCoA results with Euclidean distance (pz) and original structure with Bray-Curtis dissimilarity (px). N = 200 samples were generated. (PDF) [file pcbi.1014102.s002.pdf]

A

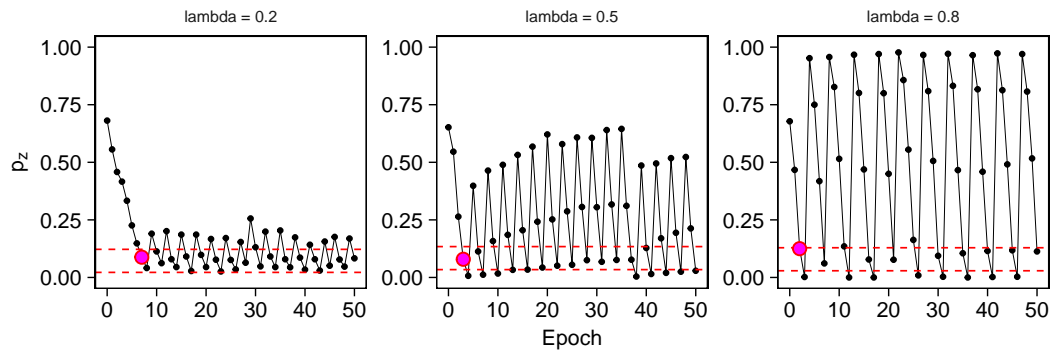

B

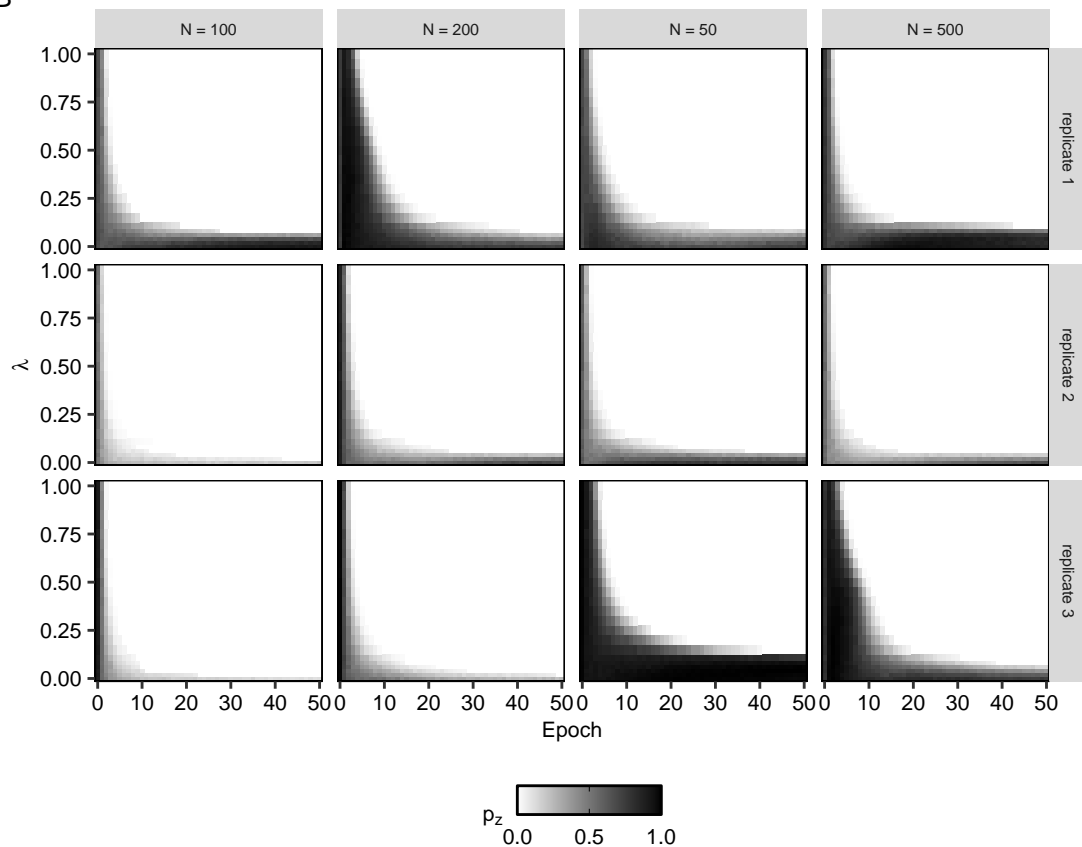

Supplement: S2 Fig — (A) Trajectories of pz across 50 epochs for λ=0.2,0.5, and 0.8. Red dashed lines indicate the region of validity (|pz−px|<0.05). Pink points highlight where the stopping rule triggers, capturing the optimal statistical alignment before further oscillations occur. (B) Trajectories of p-values with the stopping rule (|pz−px|<0.05). Heatmaps show pz values plotted against training epochs for hyperparameters λ∈[0,1]. Triplicate datasets of varying sizes (N = 50, 100, 200, 500) were evaluated to demonstrate consistent stabilization across conditions. (PDF) [file pcbi.1014102.s003.pdf]

**A**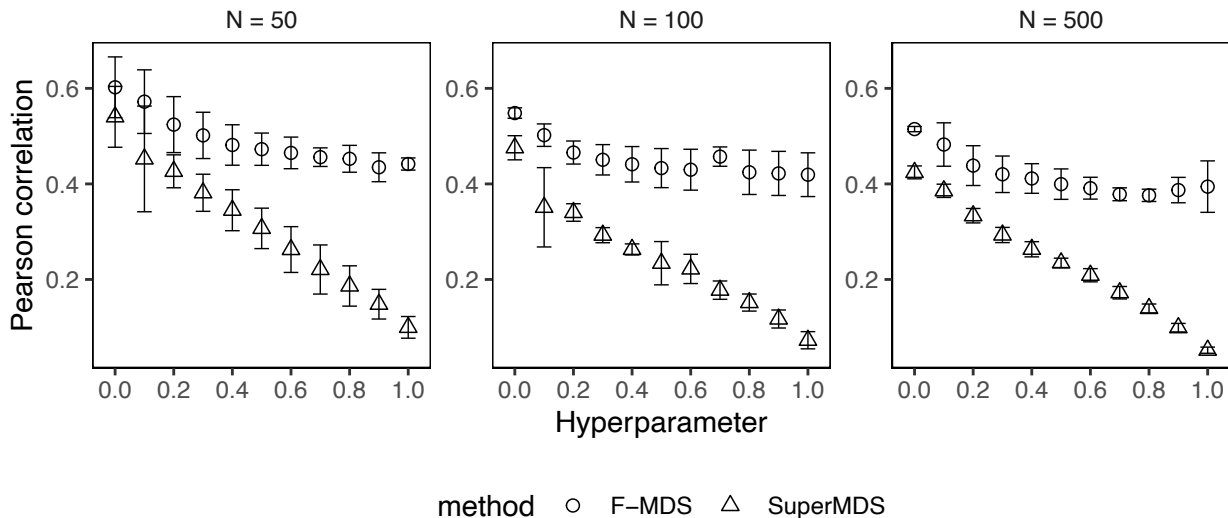**B**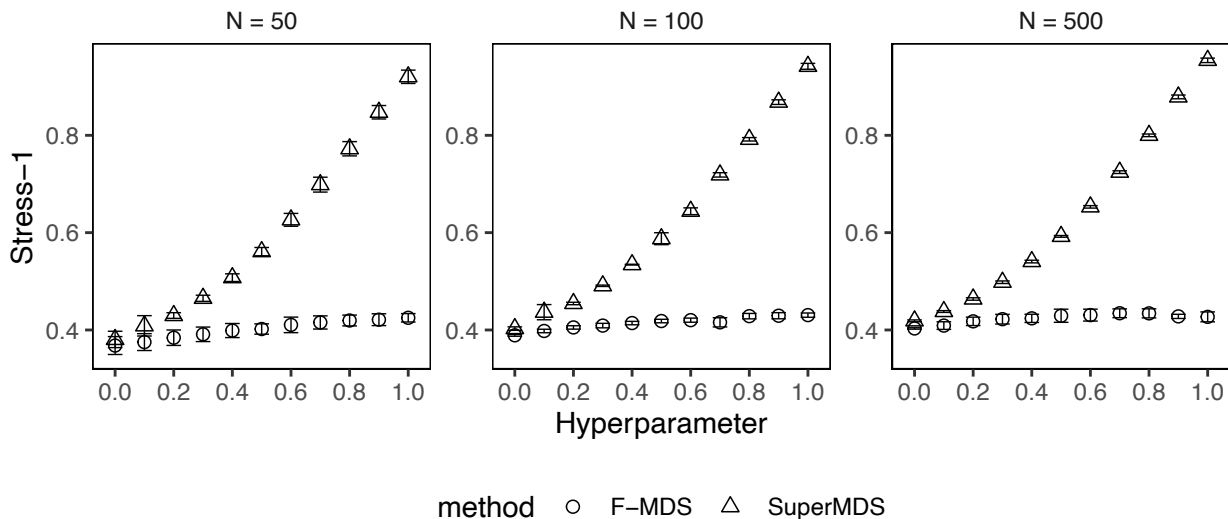

Supplement: S3 Fig — The plots are titled with the dataset size N and compared across different methods with hyperparameter values as follows: λ, F-MDS; α, superMDS. After calculating pairwise distances with Bray-Curtis dissimilarity (original) and Euclidean (2D representation), (B) their Pearson correlation coefficient and (C) normalized stress (Stress-1) were obtained. Error bars are standard deviation of triplicates. (PDF) [file pcbi.1014102.s004.pdf]

**A**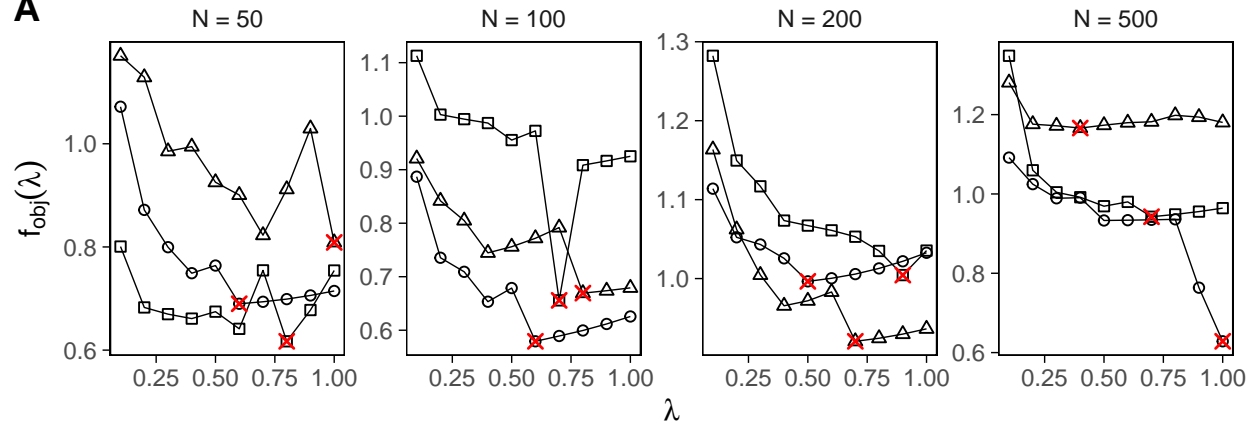**B**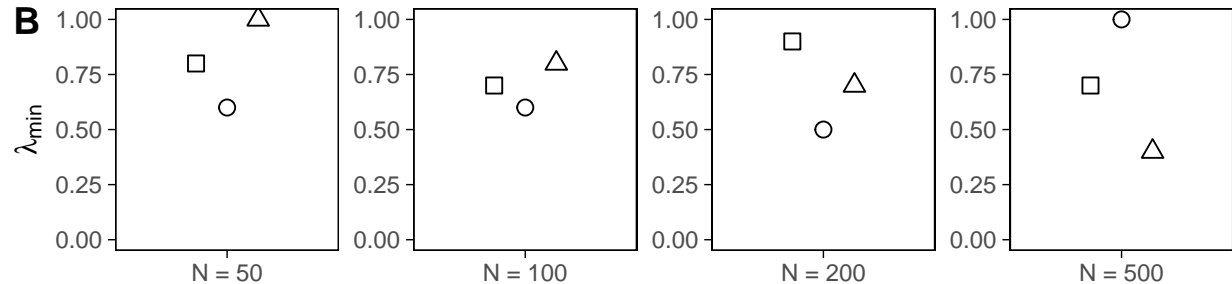

Supplement: S4 Fig — An objective function fobj(λ) was defined to simultaneously reflect the number of training epochs and the preservation of the original structure (see Equation S17). (A) For each semisynthetic dataset size N, fobj(λ) is plotted against the hyperparameter λ. The minimum value of fobj(λ) is highlighted in red. (B) The optimal hyperparameter λmin is plotted against dataset size N, with each dataset represented by a symbol. (PDF) [file pcbi.1014102.s005.pdf]

**A**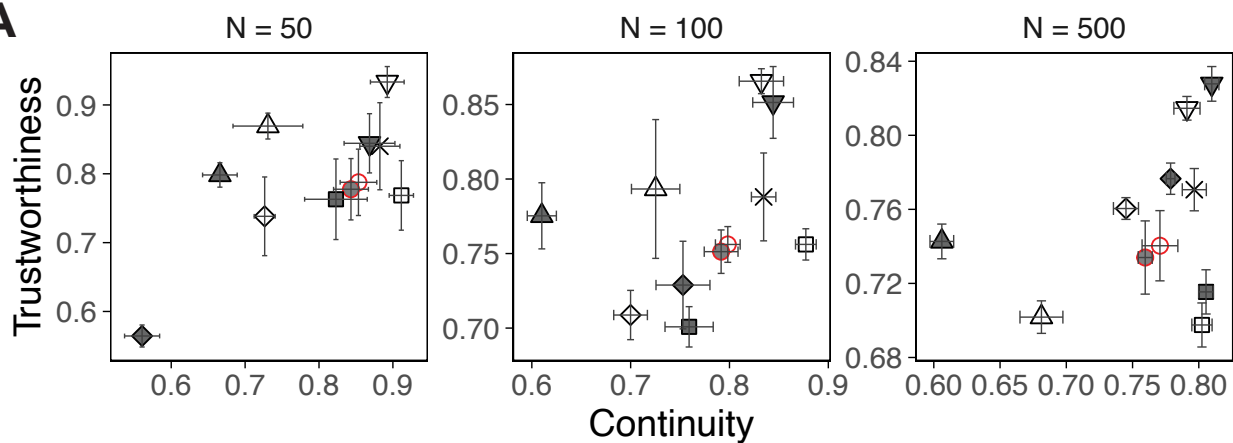**B**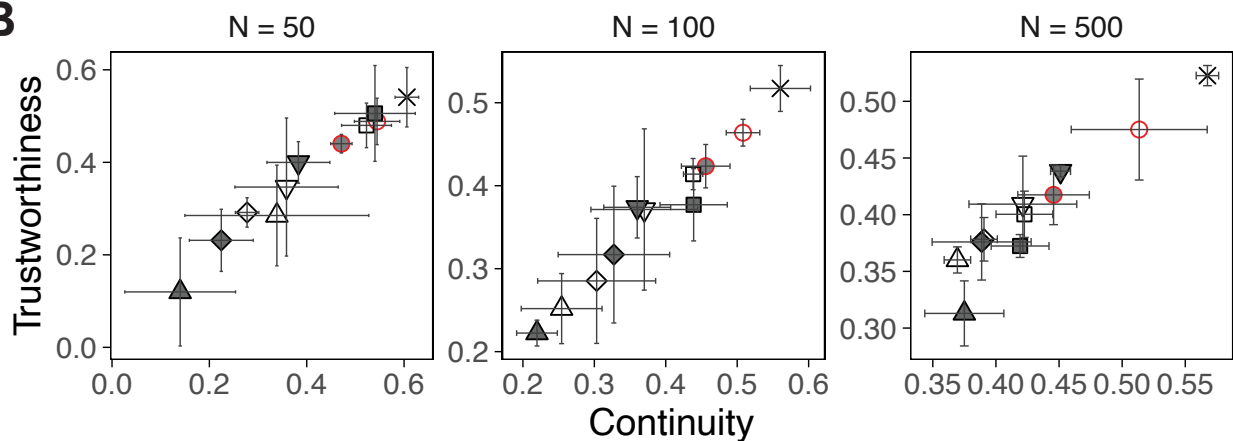**C**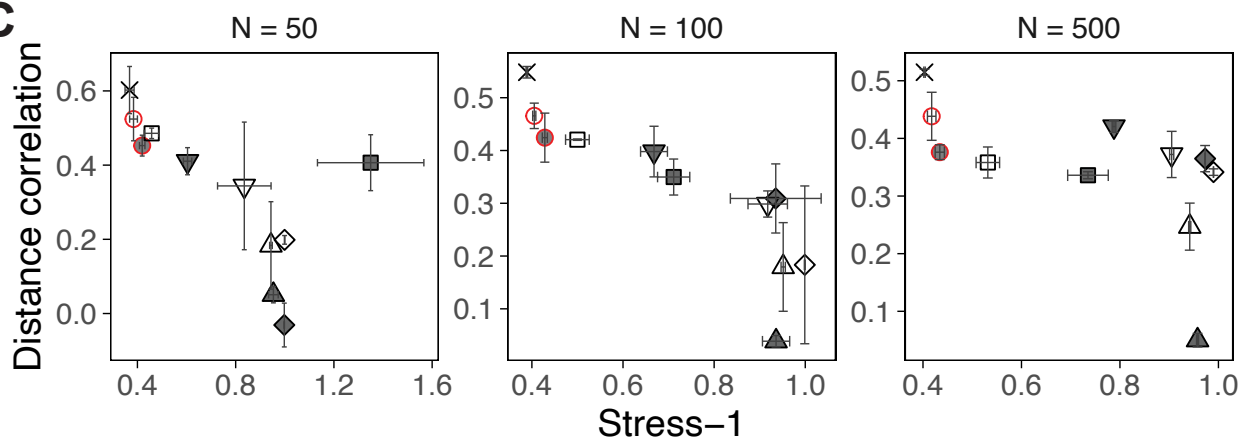**D**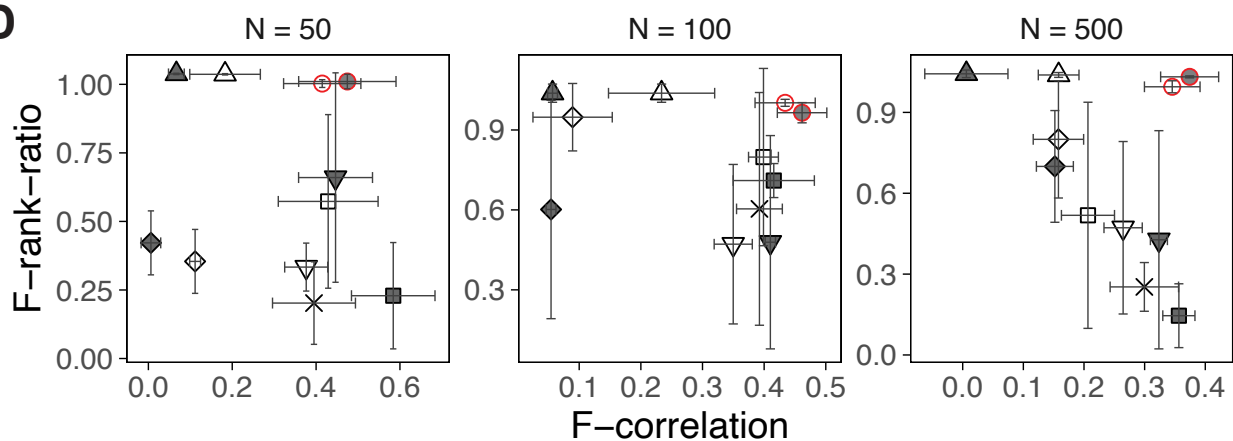

method ○ F-MDS □ Isomap × MDS ◇ t-SNE △ UMAP-S ▽ UMAP-U  
hyperparameter λ ■ 0.8 □ 0.2 n, perp ■ 50 □ 5

Supplement: S5 Fig — Trustworthiness and continuity to evaluate (A) Local structural preservation is assessed using trustworthiness and continuity. (B) Global structural preservation is similarly evaluated with trustworthiness and continuity. (C) Global distortion is quantified by Stress-1 and Pearson correlation of Shepard diagrams. (D) Preservation of statistical inference is measured by the F-rank-ratio and F-correlation using randomly permuted label sets. The following hyperparameters were used for each method: λ for F-MDS, number of neighbors n for UMAP (both supervised (-S) and unsupervised (-U)), perplexity (perp) for t-SNE, and the number of shortest dissimilarities n for Isomap. Error bars represent the standard deviation across triplicate measurements. (PDF) [file pcbi.1014102.s006.pdf]

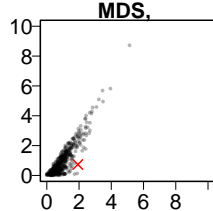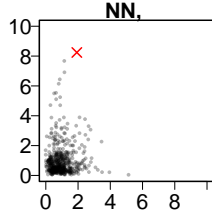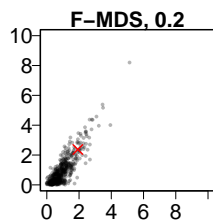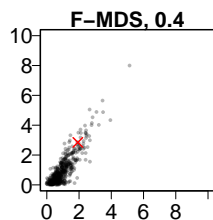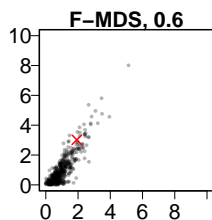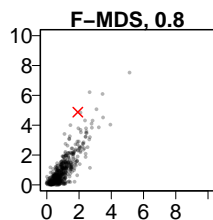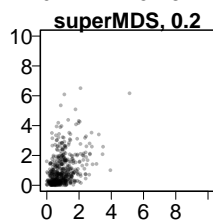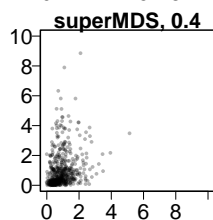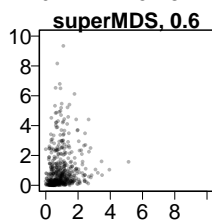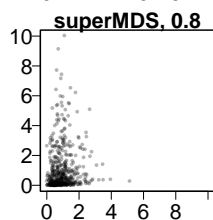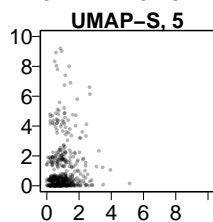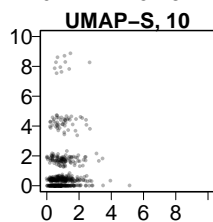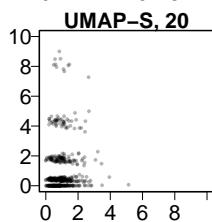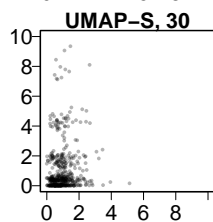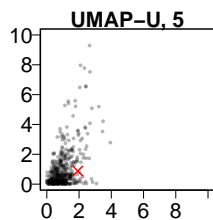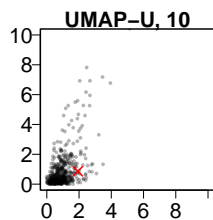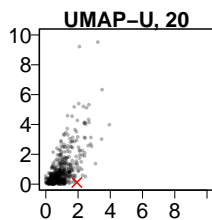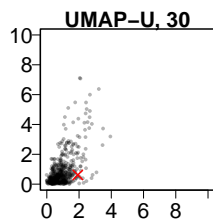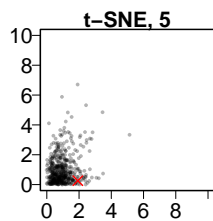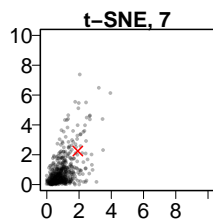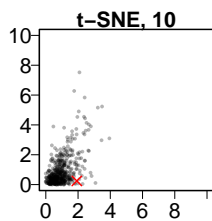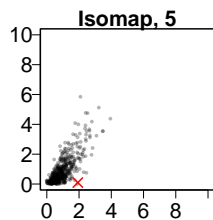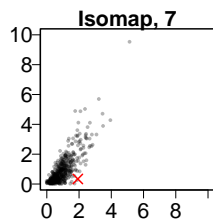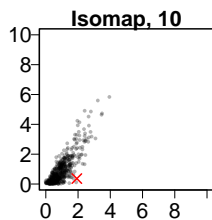

Supplement: S7 Fig — Pseudo F-ratios comparing the original dimension (x-axis) and from eight dimension reduction methods (y-axis) with algal microbiome data. Pseudo F’s were calculated by randomly permuting labels by 500 times. Highlighted with red denotes the location of F’s from unpermuted labels. Each plot is titled with the method and hyperparameter used. (PDF) [file pcbi.1014102.s008.pdf]

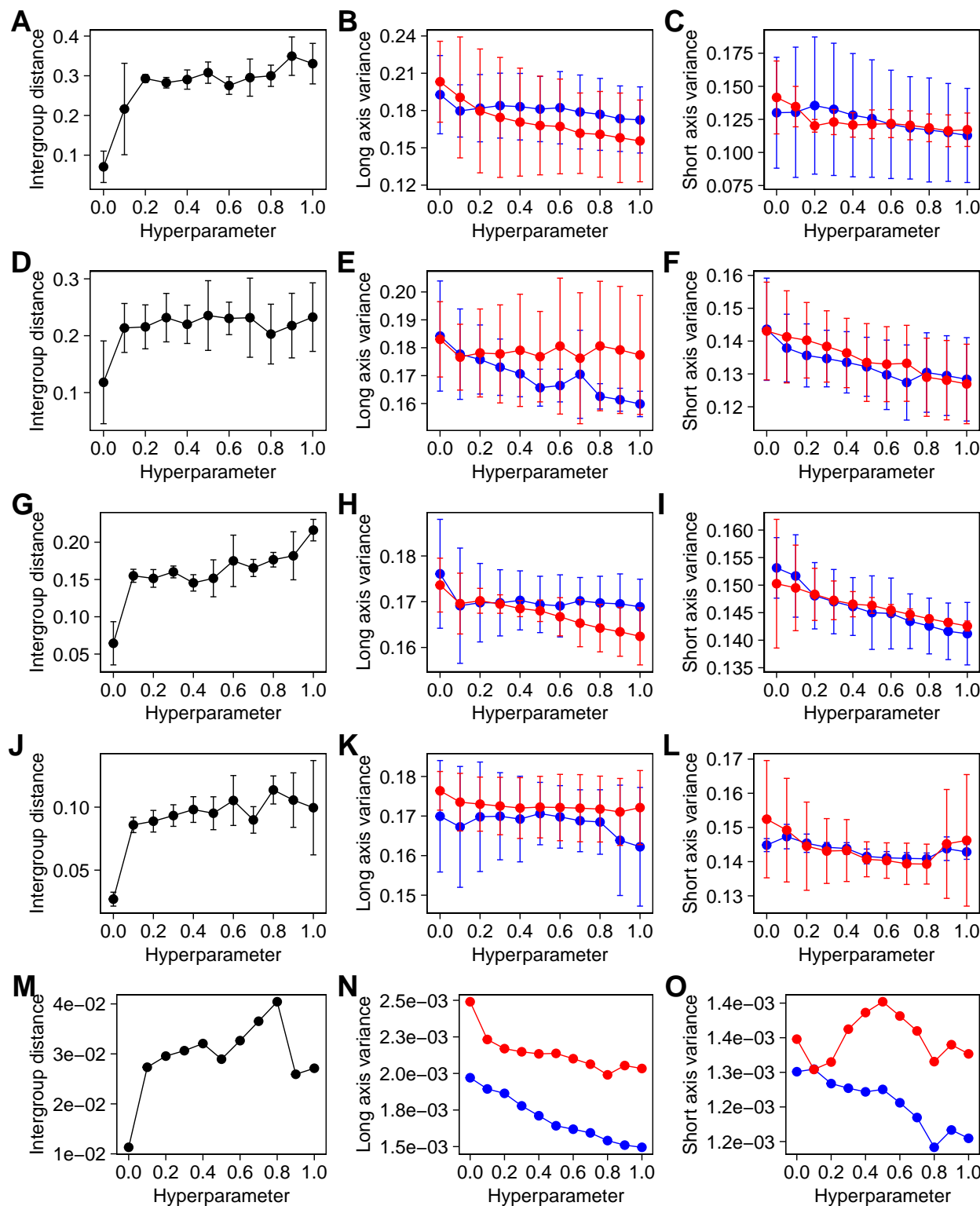

Supplement: S8 Fig — Cluster centroids and variances of F-MDS representations are shown for semisynthetic datasets of size N = 50 (A–C), N = 100 (D–F), N = 200 (G–I), N = 500 (J–L) and for the algal microbiome (M–O). The first column in each row displays the distance between group centroids. The second and third columns show the variance of each group, measured along the long and short principal axes, respectively. For the variance panels, blue and red colors denote groups 1 and 2. Error bars represent the standard deviation across triplicate measurements. (PDF) [file pcbi.1014102.s009.pdf]

**A**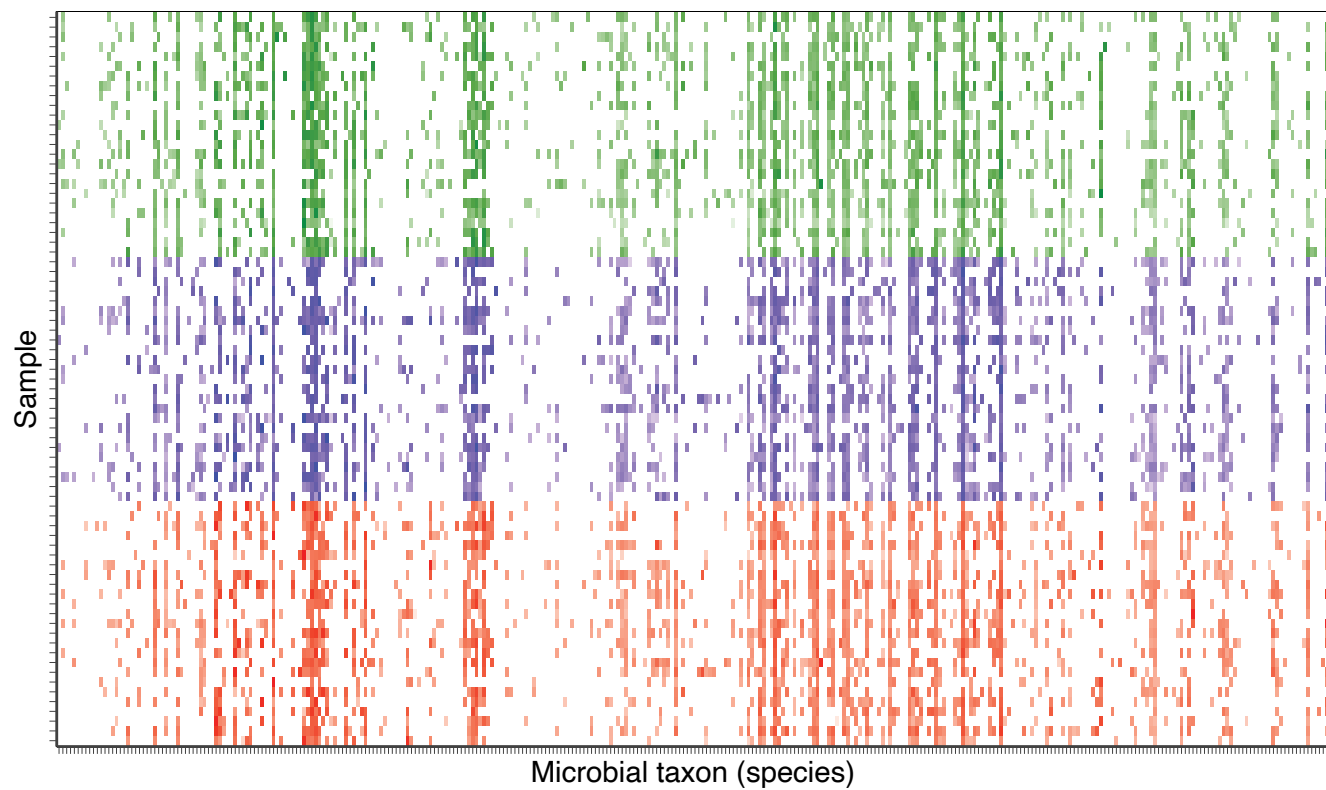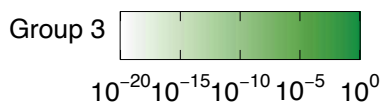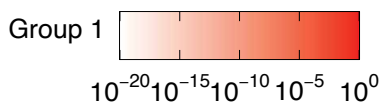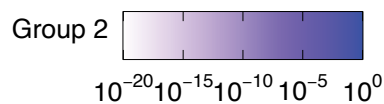**B**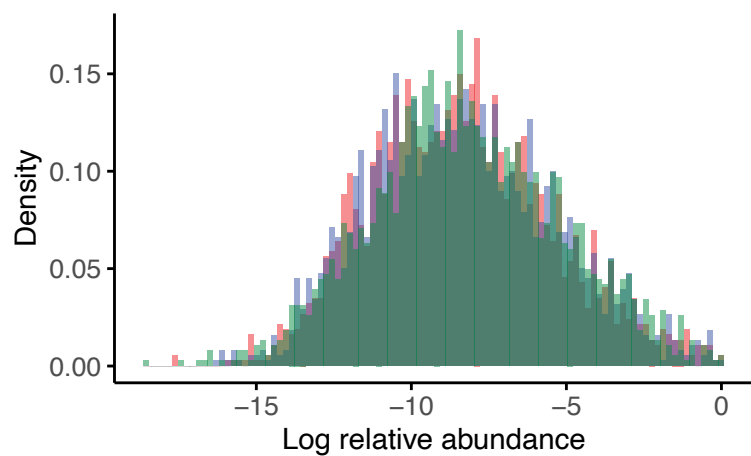

Group 1 2 3

**C**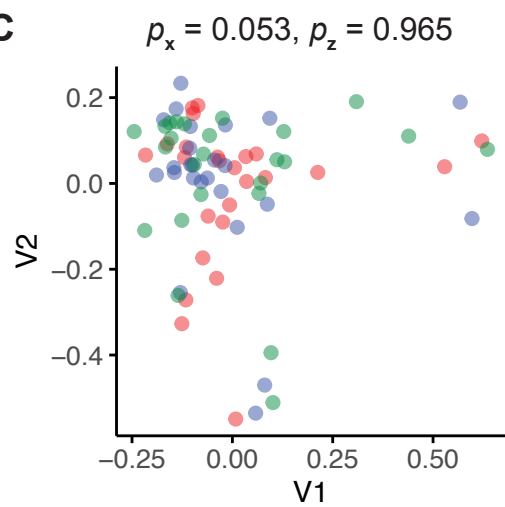

Group 1 2 3

Supplement: S9 Fig — (A) Heatmap of its log-scaled relative abundance by its microbial taxa and sample number. (B) Density histogram of the data and (C) principal coordinates analysis (PCoA) and PERMANOVA p-values based on PCoA results with Euclidean distance (pz) and original structure with Bray-Curtis dissimilarity (px). N = 75 semisynthetic data were generated. (PDF) [file pcbi.1014102.s010.pdf]
