# Supplementary material for: Multidimensional scaling informed by F-statistic: Visualizing grouped microbiome data with inference
Source: PLoS Comput Biol. 2026 Apr 1;22(4):e1014102. doi: 10.1371/journal.pcbi.1014102 (PMC13108906; doi:10.1371/journal.pcbi.1014102)
Supplement: S6 Fig — The plots are titled with the respective method and hyperparameter values as follows: λ, F-MDS; α, superMDS; Nearest neighbors number, supervised (-S) or unsupervised (-U) UMAP; Perplexity, t-SNE; Shortest dissimilarities number, Isomap; none, neural network (NN). X- and Y-axis denote distances in the original and embedding dimensions, respectively. (PDF) [file pcbi.1014102.s007.pdf]

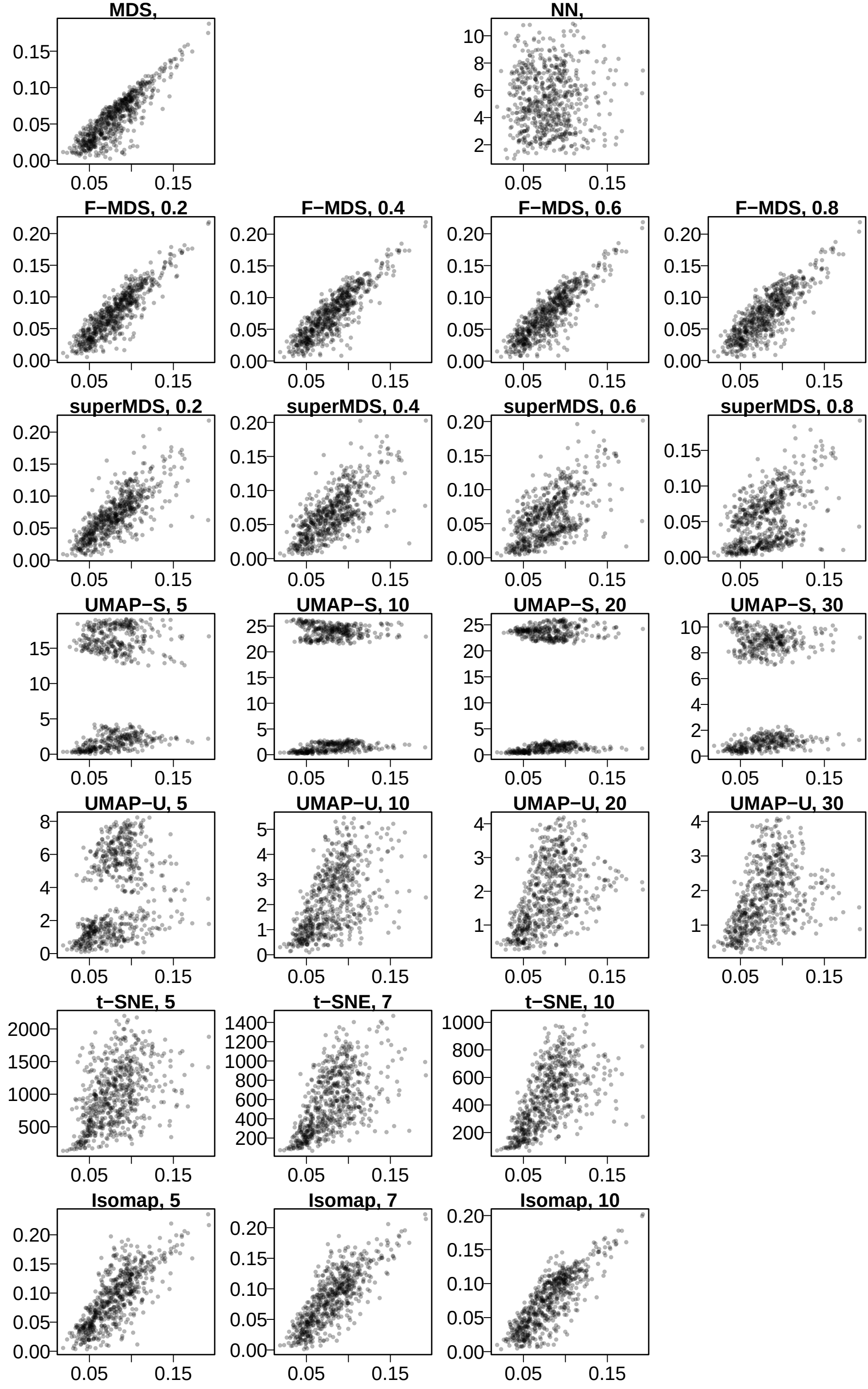
MDS,
NN,
F−MDS, 0.2
F−MDS, 0.4
F−MDS, 0.6
F−MDS, 0.8
superMDS, 0.2
superMDS, 0.4
superMDS, 0.6
superMDS, 0.8
UMAP−S, 5
UMAP−S, 10
UMAP−S, 20
UMAP−S, 30
UMAP−U, 5
UMAP−U, 10
UMAP−U, 20
UMAP−U, 30
t−SNE, 5
t−SNE, 7
t−SNE, 10
Isomap, 5
Isomap, 7
Isomap, 10
