# Supplementary material for: Multidimensional scaling informed by F-statistic: Visualizing grouped microbiome data with inference
Source: PLoS Comput Biol. 2026 Apr 1;22(4):e1014102. doi: 10.1371/journal.pcbi.1014102 (PMC13108906; doi:10.1371/journal.pcbi.1014102)
Supplement: Attachment — Submitted filename: Response to Reviewers.pdf [file pcbi.1014102.s012.pdf]

Hyungseok Kim, Ph.D.
Postdoctoral Associate
Department of Chemical Engineering
Massachusetts Institute of Technology
77 Massachusetts Ave., Cambridge MA 02139, USA
hskimm@mit.edu | (617) 780-1913

September 28, 2025

PLOS Computational Biology
San Francisco, CA, USA

Dear Editor and Reviewers,

We are grateful to the reviewers for their insightful comments, all of which we have considered carefully and addressed in the response letter. Each reviewer comment is followed by our point-by-point responses **in bold** on the subsequent pages. Below, we summarize the major changes made to the manuscript:

1. A new simulation was performed, and semisynthetic microbiome community datasets were generated using SparseDOSSA (Ma et al., *PLOS Comput. Biol.*, 2019). The new datasets were used to update the main and supplementary figures, confirming that our proposed method remains robust, consistent, and competitive in performance compared to existing methods (see Response 1-2, Response 2-3).
2. A quantitative and rational framework was introduced for hyperparameter selection using a unique grid search method (see Response 1-5).
3. The Introduction section has been revised to better describe the limitations of current MDS methods for analyzing microbiome data (see Response 2-1).
4. The software code has been updated to clarify situations in which MDS alterations are recommended. The software has now been accepted as a Bioconductor package.

We hope that our responses adequately address the reviewers' concerns and look forward to any further recommendations. Should you have any questions, please feel free to contact me.

Thank you very much.

Best regards,
Hyungseok Kim on behalf of all co-authors

Response to Comments by Referee #1

COMMENT 1-1.
The authors propose a new MDS-based ordination method, *F*-informed MDS, which incorporates the *F*-statistic to guide dimensionality reduction in microbial ecology data. This method adds a supervised component to traditional ordination, which typically does not utilize group labels. While the approach is interesting and potentially useful, I have several concerns and questions:

The method relies on predefined group labels to compute the *F*-statistic. How would the method perform when such labels are unavailable or when there is no clear group separation in the data?

RESPONSE 1-1.
First, we sincerely appreciate the reviewer's evaluation and support for our work. We apologize for not clarifying such situations in the initial manuscript, as we acknowledge that they frequently arise in real data analysis. When group separation is unclear or labels are unavailable, we maintain that metric multidimensional scaling (MDS), also known as principal coordinate analysis (PCoA), remains sufficient for the following reasons:

- **First, metric MDS / PCoA is a standard, well-established ordination method for visualizing microbiome data. Because microbiome data often exhibit distinct structures, analytical approaches tailored to microbiome studies are preferred (Peterson et al. *Annu. Rev. Stat. Appl.* 2024).**
- **Second, our quality metric analysis demonstrates that metric MDS remains competitive with benchmark ordination methods (UMAP, Isomap, t-SNE; see main Figure 4). Specifically, metric MDS best preserves global structure across four evaluation metrics. It also ranks as the second-best method for preserving local structure, exceeded only by unsupervised UMAP. Together, these results confirm that metric MDS provides a reliable representation of microbiome community data.**
- **Third, when group separation is absent in the original data, it may be challenging to extract biological insights from ordinations (differential abundance analysis is recommended instead). Ordination methods display overall data distributions, whereas differential analysis operates feature-wise, offering greater potential for biological discovery in such cases.**
- **Finally, when labels are unavailable, direct statistical inference is challenging. However, biological implications can still be inferred through latent variable modeling of covariances, as demonstrated by Shi et al. (*Bioinformatics*, 2020).**

In the Main Text, we have clarified how our method will work when there is no group separation. They are now shown in Figure 1, lines 81-83 of page 4 (Introduction), and in lines 175-178 of Page 7 (Methods) of a marked-up copy version, which read as:

> "…We applied the perturbation only to datasets for which metric MDS failed to detect a statistically significant group-wise difference under the original data structure." (lines 81-83)

"… It should be noted that the update is applied exclusively to the representation where its group-wise difference is not identified ($p_z \ll p_x$), which is significant under the original structure ($p_x < 0.1$)." (lines 175-178)

We have also updated our software which is now available in Bioconductor, by clarifying if-else statement that determines if input labels are absent or group difference is not statistically significant.

COMMENT 1-2.
As noted earlier in the manuscript, microbiome data are typically compositional and often zero-inflated. However, it appears that the simulated data used to validate the method did not account for these features.

RESPONSE 1-2.
We thank the reviewer for raising this important point about the need for simulation that address the unique challenges of microbiome data. We have carefully considered this comment and made significant changes as below.

1) **Simulation model selection: We referred to a literature review that identified statistical methods for generating realistic microbiome datasets (Sankaran et al., Brief. Bioinform. 2025). Among the 11 available models, we selected SparseDOSSA, as it explicitly models data with both compositionality and zero-inflation through a zero-inflated log-normal distribution and L1 normalization (Ma et al., PLOS Comput Biol. 2021). Of note, the performance of SparseDOSSA has been validated by direct comparison to real microbial community profiles, confirming its ability to generate realistic synthetic datasets.**
2) **Dataset generation: We then generated datasets using parameters estimated from human stool microbiome samples, featuring 332 taxa. Our primary simulation goal was to demonstrate examples where conventional metric MDS fails to reveal group structure in two-dimensional ordination, while *F*-informed MDS, by contrast, can recover such structure. After model fitting, we adjusted the group means and generated semi-synthetic datasets with statistically distinguishable group structures. With sample sizes ranging 50-500, these modifications ensured both zero-inflation and the desired degree of group separation.**
3) **Method evaluation: Finally, we applied the *F*-informed MDS and existing ordination methods to the new synthetic datasets. We observed that, with these zero-inflated and compositional data, the proposed method still remained robust, consistent, and competitive in performance compared to existing methods.**

The revised simulation settings are described in lines 194-232, pages 8-9 (Methods) and lines 286-301, page 11 (Results) in the marked-up copy version. Moreover, the simulation results and their evaluation have also been newly updated. They are now shown in main Figures 2-4, 6A, 7, and Supplementary Figures 1-5, 8, 9.

COMMENT 1-3.
The simulations assume a diagonal covariance matrix, implying no correlation among microbial features. In reality, microbial taxa often exhibit complex interactions—both positive and negative correlations. This simplifying assumption may limit the generalizability of the method's performance in real-world data.

RESPONSE 1-3.
We again appreciate reviewer's comment on the simulation settings. The newly adopted simulation model for our revised manuscript, SparseDOSSA, does capture a correlative characteristic between microbial features. This is achieved by indirectly expressing the correlation through a copula parameter that describes a latent variable determining whether a microbial feature is present (i.e., sparsity). Of note, its model publication, SparseDOSSA 2, have shown ability to exhibit "feature-feature correlations" (Ma et al., *PLOS Comput Biol.* 2021).

In our revised manuscript, we describe how our choice of simulation model implies microbial feature correlation in Methods section which reads as:

> "… In addition, dependency among features in SparseDOSSA is indirectly handled by the copula parameter $\Omega \in \mathbb{R}^{S \times S}$. This parameter describes a latent multivariate Gaussian variable that is compared with π_s to determine whether the feature is present." (lines 207-210)

COMMENT 1-4.
Microbiome datasets are frequently high-dimensional. It is unclear how the proposed method performs in such scenarios. Are there any computational or numerical stability issues when the number of features exceeds the number of samples?

RESPONSE 1-4.
In our new simulation, we generated several semi-synthetic datasets, including cases where the number of features exceeded the number of samples. Specifically, the simulations involved reducing 332 dimensions (i.e., microbial features) into 2D representations with sample sizes of N = 50, 100, 200 and 500. In all cases, we did not observe any computational or numerical stability issues. These empirical findings suggest that our algorithm optimizing the *F*-MDS objective function with Stress majorization, is stable for microbiome data representation indeed.

In general, the stress majorization procedure, known as SMACOF, is monotone non-increasing and converges to fixed points provably for the MDS objective function (De Leeuw, arXiv, 2024). However, it can suffer from computational complexity, especially with high-dimensional data. Whether our algorithm employing SMACOF guarantees monotonicity and convergence of the *F*-MDS objective remains to be fully explored. Alternatively, gradient descent–based optimization methods can mitigate computational challenges (Zheng et al. TVCG, 2017), but they do not guarantee stability and remain an active area of research for improvements (Fang et al., AISTATS 2025).

Reflecting these new literature findings, we have newly added sentences in paragraph 2 of Discussion section which now reads:

> "… Indeed, majorization for MDS has been known for its high computational cost [48], although its numerical convergence is guaranteed [71] and we did not observe any stability issues in our simulation. Recent theoretical advances in computational algorithms, including majorization [72] or (stochastic) gradient descent [39,40] open doors for improving our framework toward unrestricted initialization and more efficient iterations." (line 512-514 in marked-up copy)

COMMENT 1-5.
The manuscript explores the effect of different λ values through a grid search. Would it be possible to adopt a cross-validation framework to select λ in a more scientific approach?

RESPONSE 1-5.
We wholeheartedly agree that λ should be chosen in a more scientific approach. Cross-validation is indeed a widely accepted procedure for hyperparameter optimization in standard machine learning methods. However, in our applications to F-informed MDS, we believe that cross-validation may not be the most suitable approach for the following reason. We propose an alternative framework instead.

1) **Although an objective function is defined and minimized at each epoch like other machine learning methods, we do not use this function as a proxy to monitor *F*-MDS performance. This is because, as shown in main Figure 2, *F*-MDS objective terms at convergence generally do not scale with λ. In other words, minimizing the objective function does not necessarily indicate the optimal λ. Instead, we use *p*-values as an alternative proxy to assess the validity of *F*-MDS. This differs from typical machine learning models, where accuracy or error rate explains classification / regression performance and are influenced by hyperparameter tuning.**
2) **Our grid search with varying λ values indicates that λ influences (i) the number of computational steps required for convergence, and (ii) preservation of pairwise distance. These effects are described using epoch number n_{epoch} and Pearson correlation ρ_{dist} (main Figure 2C and 3B, respectively). Reflecting this, we newly introduce a quantitative framework for hyperparameter selection. The optimal λ is defined as the minimal value allowing the epoch number to reach its lower bound.**
3) **t should be noted that cross-validation typically involves repeatedly validating a trained model on subsets of the data. Ordination methods, however, primarily focus on finding a representation of a single dataset. The resulting representation is not a predictive model and is not intended for application to other datasets or validation sets. Although cross-validation may be feasible with sufficiently large datasets (e.g., by *k*-fold splitting), we believe it is unnecessary and overkill in this context.**

Accordingly, we have newly added text in Results section, new S3 Appendix, and Supplementary Figure 4 to describe our hyperparameter selection framework:

> "… To select the hyperparameter quantitatively, a grid search was conducted (see S3 Appendix). We introduced an objective function $f_{obj}(\lambda)$ that balances the increased computational cost against increased structural deviation, evaluating this function over λ in [0, 1] (S4 Fig A). For all semisynthetic datasets, the hyperparameter λ that minimized $f_{obj}(\lambda)$ was greater than or equal to 0.5 (0.78 ± 0.17, mean ± s.d.; see S4 Fig B). This result confirms that the performance of our selection framework is largely determined by the number of computation epochs." (line 344-350 in marked-up copy)

COMMENT 1-6.
How does F-informed MDS behave when there is no actual difference between the groups (i.e., under the null)? Does it still produce a stable embedding?

RESPONSE 1-6.
Similar to our response to the Comment 1-1, we believe that metric MDS is sufficient when there is no actual group difference. In the newly submitted manuscript and its software, we introduced if-else condition that sets a rule dictating when *F*-informed MDS will operate. Again, the scope of data conditions which *F*-MDS is recommended has been clarified in lines 81-83 and lines 175-178 of a marked-up copy version.

Response to Comments by Referee #2

In this study, Kim and colleagues introduce a multidimensional scaling (MDS) method based on an F-statistic for grouped microbiome data. However, I think the authors overstate the importance of this method and the potential of using this method for analyzing and interpreting microbiome data.

COMMENT 2-1.
The proposed approach is designed to preserve the hypothesis testing output for group differences between the original dataset and the MDS representation. However, the authors do not provide any explanations or reasoning (Lines 107–152) regarding why the significance of group differences between the original dataset and the MDS representation should be similar. For example, if group differences are significant in the original dataset but not in the MDS representation, does this imply that the MDS representation is not reliable? A significant group difference in the original dataset could be due to dimensions beyond the 2-dimensional MDS, which are not captured in the MDS representation.

RESPONSE 2-1.
We greatly appreciate the reviewer's point and apologize if our explanation caused any confusion. It is correct that we did not directly explain why the group difference should be reflected in the MDS representation. Explicitly, the way MDS computes the dataset does not take group differences (e.g., labels) into account, so there is no inherent reason MDS representation should always display the group difference. Implicitly, if the structures are different – and only if the difference is significant enough to be well visualized under lower dimensions (which can be two or more) – then yes, MDS is likely to reflect the group difference.

The situation we address is when the group difference is statistically significant but not well visualized through MDS ordinations. Scree plot analysis can suggest the dimensionality at which the difference may be visualized. However, it does not inform us about how samples are distributed in such dimensions, particularly if the dimensionality exceeds three, and standard MDS method cannot directly resolve this challenge. While such situations may be outliers and are not universally observed in real microbiome datasets, they do exist and remain unresolved.

It is also noteworthy that MDS was first formally developed in 1950s, when data types were much less complex than modern microbiome communities. Considering the advancements in microbiology, we believe improvements in MDS are imperative.

We have added sentences to the main text, clarifying current challenges with MDS visualization for cases where groups are statistically different but not well visualized. These additions appear in paragraph 4 of the Introduction as:

> "… To test for structural change and to correlate results with treatment effects, an additional and independent differential abundance analysis is required [5,6]. While metric MDS can detect some changes [33], its low-dimensional representation does not always

capture every underlying pattern—even when statistical validation confirms the presence of such a pattern in the original space. A pattern is pronounced in the low-dimensional embedding only if the distance structures between groups are sufficiently distinct to allow a clear visualization. When this is not the case, scree plot analysis can be used to determine the dimensionality at which the pattern becomes visible. However, a scree plot does not indicate how the samples are distributed within this reduced dimension. Moreover, if the required dimensionality exceeds three, metric MDS cannot effectively visualize the difference between groups." (lines 48-62, marked-up copy version)

COMMENT 2-2.
A second major concern is the authors' claim that the proposed method is specifically designed for compositional data in microbiology and ecology. There is no clear connection between compositional data analysis and the proposed method since it only requires a distance matrix between samples.

RESPONSE 2-2.
We agree with the reviewer's point that the original claim lacked strong logic. We apologize if certain topics in this manuscript did not adequately address current challenges in compositional data analysis (CoDA). Our newly generated simulation data now exhibit major characteristics emphasized in CoDA, such as zero-inflation and feature-wise covariance structure (for details, please see RESPONSE 2-3). Additionally, we highlight the computational rigor of our algorithm when analyzing these datasets (e.g., paragraph 2 of the Discussion).

We have also revised sentences that may previously have implied a direct connection between CoDA and microbiome data analysis. Including in the Abstract, the use of the word "compositional" has now been specifically reduced from 12 to 6 occurrences in the main text.

COMMENT 2-3.
A third major concern is that the simulation settings (Lines 154–168) are too simplistic to demonstrate the usefulness of MDS methods. In the simulation studies, the dimension of the original dataset is only 4, and the degrees of freedom are reduced to 3 due to the compositional constraint. Why not plot the scatter plots 3 of 4 for all variable pairs in this case?

RESPONSE 2-3.
We thank the reviewer for raising this important point about the need for simulation that address the unique challenges of microbiome data. We have carefully considered this comment and made significant changes as below.

1) **<u>Simulation model selection</u>: We referred to a literature review that identified statistical methods for generating realistic microbiome datasets (Sankaran et al., Brief. Bioinform. 2025). Among the 11 available models, we selected SparseDOSSA, as it explicitly models data with both compositionality and zero-inflation through a**

zero-inflated log-normal distribution and L1 normalization (Ma et al., PLOS Comput Biol. 2021). Of note, the performance of SparseDOSSA has been validated by direct comparison to real microbial community profiles, confirming its ability to generate realistic synthetic datasets. For example, the model captures a correlative characteristic between microbial features by indirectly expressing the correlation through a copula parameter.

2) **<u>Dataset generation</u>: We then generated datasets using parameters estimated from human stool microbiome samples, featuring 332 taxa. Our primary simulation goal was to demonstrate examples where conventional metric MDS fails to reveal group structure in two-dimensional ordination, while *F*-informed MDS, by contrast, can recover such structure. After model fitting, we adjusted the group means and generated semi-synthetic datasets with statistically distinguishable group structures. With sample sizes ranging 50-500, these modifications ensured both zero-inflation and the desired degree of group separation.**
3) **<u>Method evaluation</u>: Finally, we applied the *F*-informed MDS and existing ordination methods to the new synthetic datasets. We observed that, with these zero-inflated and compositional data, the proposed method still remained robust, consistent, and competitive in performance compared to existing methods.**

The revised simulation settings are described in lines 194-232, pages 8-9 (Methods) and lines 286-301, page 11 (Results) in the marked-up copy version. Moreover, the simulation results and their evaluation have also been newly updated. They are now shown in main Figures 2-4, 6A, 7, and Supplementary Figures 1-5, 8, 9.

COMMENT 2-4.
Additionally, there appear to be typos in Equations (9) and (10) on Page 8. In Equation (9), "N" should be the variable dimension 4, and in Equation (10), "x_i" should be normalized to reflect the compositional nature of microbiome data, where relative abundances are measured and the count matrix from sequencing technologies should be normalized to sum to 1 in each sample.

RESPONSE 2-4.
We appreciate the reviewer's detailed attention pointing out the typos in Equations and suggestions to be made. They have now been fixed or been replaced with new expressions describing the semisynthetic dataset.
