# Supplementary material for: Multidimensional scaling informed by F-statistic: Visualizing grouped microbiome data with inference
Source: PLoS Comput Biol. 2026 Apr 1;22(4):e1014102. doi: 10.1371/journal.pcbi.1014102 (PMC13108906; doi:10.1371/journal.pcbi.1014102)
Supplement: Attachment — Submitted filename: Response to Reviewers_DEC2025.pdf [file pcbi.1014102.s013.pdf]

Massachusetts Institute of Technology

Cullen R. Buie, Ph.D.
Professor

December 31, 2025

PLOS Computational Biology
San Francisco, CA, USA

Dear Editor and Reviewers,

We are again grateful to the reviewer for the comments, all of which we considered carefully and responded to in this letter. We have addressed each reviewer comment **in bold** with our point-by-point responses on the following pages. Below we summarize changes that have been made in the manuscript.

1. Majorization-minimization algorithm for F-MDS was revised to compute F-ratios using the same set of permutations in both dimensions (Response 3-1).
2. Stopping rule for the algorithm was specified in the main text (Response 3-2 (iii)).
3. Hyperparameter selection procedure was revised and its results were updated (Response 3-2 (iv))

We hope that the responses are satisfactory and look forward to publication. Should you have any question, please feel free to contact me.

Thank you very much.

Best regards,

Cullen R. Buie

Mechanical Engineering
Building 3-248

77 Massachusetts Avenue
Cambridge, MA 02139-4307

Direct: 617-253-9379
Email: crb@mit.edu
http://web.mit.edu/~lemi/

Response to Comments by Referee #3

COMMENT 3-1.

You defined the regression function f_z to link F_x^{Π} and F_z^{Π}. However, in Algorithm 1, the data pair used is ($F_x^{\Pi 1}$, $F_z^{\Pi 2}$), meaning that F_x and F_z are computed from different permutations. In that case, why should a reasonable regression function f_z exist between them? For instance, it would be analogous to expecting a regression relationship between a sample u ~N(0,1) and another independent sample ~N(1,1), which clearly have no meaningful correspondence.

RESPONSE 3-1.

We appreciate the reviewer's thoughtful comments on our algorithm. In response to this comment, we evaluated whether the regression function f_z can be obtained from the *F*-ratios simultaneously using a single permutation set, instead of computing the ratios using two independent permutations. When the regression functions were compared using a semisynthetic dataset of $N = 200$, no significant change was observed (see panel A of the figure below), suggesting that a single set of permutations is sufficient to link the *F*-ratios. Note that it is still necessary to sort F_x^{Π} by magnitude (and similarly for F_z) prior to performing the regression, in order to assess the significance of the original *F*-ratio and accurately map the ratio in the two-dimension (see panel B of the figure for comparison). Figure 1 is copied below for your convenience.

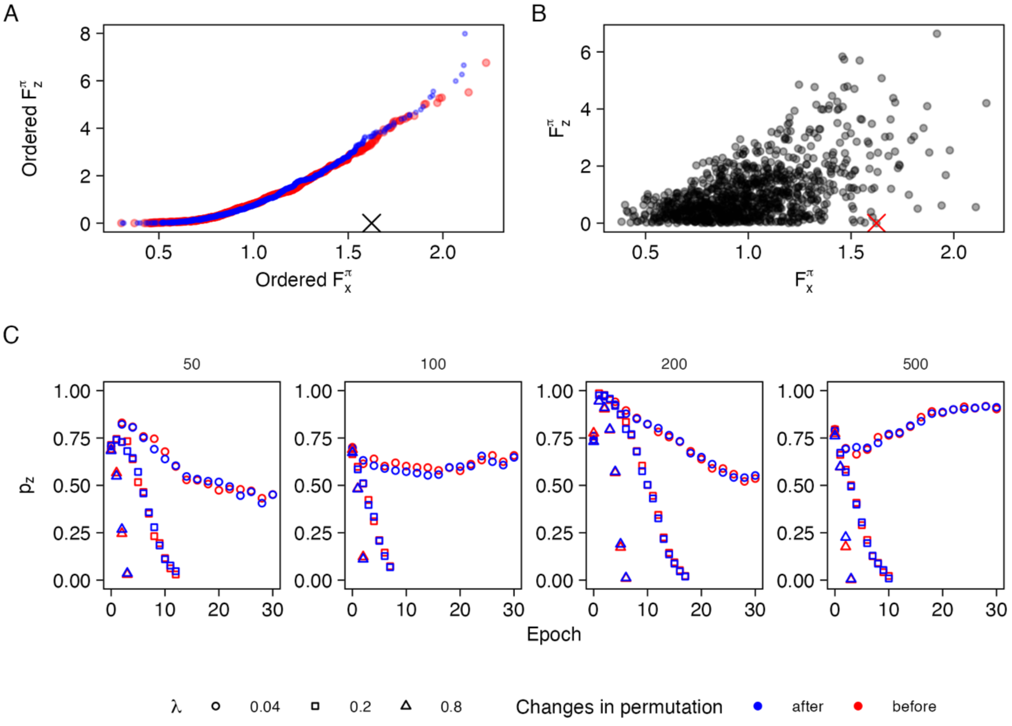

Figure 1. (A) Regressions were performed using two individually sorted *F*-ratios (F_x^{Π}, F_z^{Π}) and compared to each other based on different permutation approaches (two sets-approach, red; single set, blue). (B) Distribution of paired, unsorted *F*-ratios computed in each dimension (X and Z) using permuted labels. Original *F*-ratios from unpermuted labels set are denoted as cross mark. (C) PERMANOVA *p*-values computed in Z-space over algorithm epochs, before (red) and after (blue) applying the single-set permutation approach.

Similarly, when applying the single-set permutation approach, we observed that iteration results from the majorization algorithm (e.g., main Fig 2B) produced almost identical *p*-values (see panel C). Hence, we conclude that our proposed MDS method are not affected by changes in the label permutation scheme used for computing f_z.

In response to the reviewer's comment, we have revised our algorithm by applying the same set of permutations to the *F*-ratios. The change is shown in Algorithm 1 and S1 Appendix of the marked-up copy. Additionally, to clarify the necessity of the sorting procedure, we have revised Methods section which now reads as:

> "… In addition, prior to the regression, each ratio was computed iteratively using a set of permuted labels $y^{\Pi k}$ for $k = 1, \dots, K$, and the resulting values were sorted by magnitude. This enabled the original *F*-ratio (from the unpermuted labels) to be mapped precisely in the two-dimensional space." (lines 148-151)

COMMENT 3-2.
(i) Is it correct to say that a smaller value of λ leads to better preservation of the original distance structure, whereas a larger λ places more emphasis on preserving group differences? (ii) Equation (S17) is used as the objective function to select the optimal hyperparameter λ. However, it is rather unconventional to incorporate computation time as an explicit component of the loss function. Could you clarify the underlying principle or rationale for determining the optimal value of λ? (iii) For a fixed λ, the stopping criterion of the algorithm is not clearly specified. As suggested in Fig 1B, the procedure terminates when p_x is not substantially smaller than p_z. Could you clarify the exact rule — for instance, $| p_z - p_x | / p_x < \varepsilon$? Furthermore, if the algorithm were allowed to continue, would p_z continue to decrease toward zero, fluctuate, or converge to p_x? This behavior is not clearly reflected in the reported results (e.g., Figs. 2 and S2). (iv) This question is related to (i), (ii) and (iii). Is it correct to say that, for a given λ, the algorithm either fails to converge or that p_z always converges to p_x? Furthermore, is there a threshold value of λ such that, for values below this threshold, the algorithm does not converge, while for values above it, p_z converges to p_x? If so, would it be reasonable to select the optimal λ as this threshold?

RESPONSE 3-2.

(i) It is correct that a smaller hyperparameter value helps preserve the original distance structure. A larger λ, on the other hand, causes the 2D representation to drift from the original but ensures that the MDS highlights group differences (details to follow).

(ii) We agree that it is not conventional to use the number of epochs when defining a loss function. We previously chose the number of epochs as a representative characteristic that benefits from larger λ while sacrificing the preservation of the original structure. Other characteristics, such as group mean differences (S8 Fig), also increased with higher λ; however, the correlation was not strong (*P* = 0.078, t-test), suggesting that the intergroup distance may not be a robust metric for defining a loss function.

(iii) We apologize for not specifying the exact stopping rule in the main text — it was already implemented in Bioconductor, our published R package

(DOI:10.18129/B9.bioc.FinfoMDS). The procedure was designed to terminate at epoch t = T such that | $p_z(T) - p_x$ | is minimal and less than ε = 0.05 (see code below). The stopping rule has now been clarified in the main Fig 1B and Algorithm 2. Moreover, once the algorithm was allowed to continue, p_z values oscillated between 0 and 1, with a higher rate when λ increased. Figure 2 is copied below for your convenience.

```
fmds <- function(D, y, X, nit = 100, lambda = 0.5, threshold_p = 0.05, z0 = NULL){
(…)
        if((abs(p_up-p0) >= abs(p_prev-p0)) & (abs(p_prev-p0) <= threshold_p)){
            message(sprintf('Lambda %.2f ...halt iteration', lambda))
            z_up <- z_prev # revert to prev
            break
        }
```

Code lines of R package `FinfoMDS` where stopping rule is applied (highlighted).

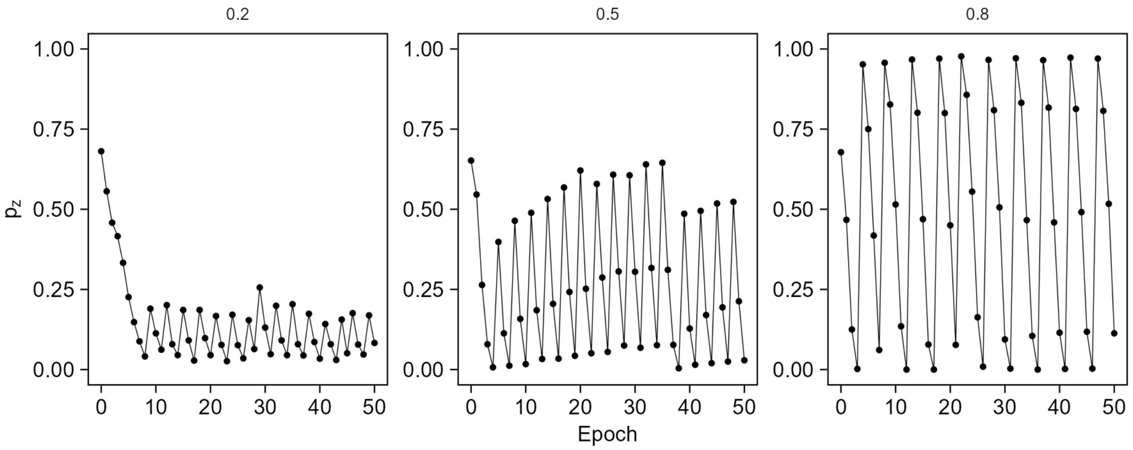

Figure 2. Changes in the PERMANOVA *p*-values over the number of *F*-MDS training epochs without stopping rule with three hyperparameter settings (*λ* = 0.2, 0.5, 0.8).

We have now reported this behavior and specified the stopping rule in paragraph 2 of Results (copied below), S2 FigA, and S2 Appendix.

"… When training continued without a stopping rule, p_z-values oscillated between 0 and 1, with a higher oscillation rate at larger *λ* (S2 FigA). The stopping rule was therefore defined to trigger once p_z approached p_x closely and their difference was minimized (S2 Appendix)." (lines 276-280)

(iv) Yes, it is correct that, for a given λ, the algorithm either failed to converge or p_z always converged to p_x. For λ less than 0.15, p_z did not converge to p_x until fifty computational epochs for at least one simulated dataset. For λ greater than unity, the algorithm did not converge, or it was not examined, because the majorized objective function (Equation S13) was no longer convex and caused the solution to diverge. For λ between 0.15 and 1, all p_z values converged to p_x in fewer than fifty epochs.

In response to the reviewer's comment, we have revised our procedure for selecting the optimal hyperparameter. Specifically, multiple quality metrics were considered to define the loss function for hyperparameter selection. These include the

hyperparameter range guaranteeing convergence, the pairwise distance correlation, and the convergence rate by epochs. To remove bias among these metrics, we now apply min–max normalization and redefined the loss function. The optimal hyperparameter is now calculated as 0.725 ± 0.186 (compared to 0.78 ± 0.17 in the previous version, mean ± s.d.). Still, no statistically significant relationship was found between the optimal λ and data size, suggesting the main conclusion was not affected by the revised procedure.

The new selection procedure is described in paragraph 5 of Results (copied below), S3 Appendix, and updated results are shown in S4 Fig.

> "… Based on the hyperparameter λ's influence on the MDS configuration, a grid search was conducted to identify an optimal value. Prior analysis demonstrated a faster *F*-MDS convergence with larger λ (Fig 2C). To balance the convergence rate against structural deviation while mitigating bias across the metrics, we defined an objective function $f_{obj}(\lambda)$ via min-max normalization (S3 Appendix). The loss function was evaluated over λ between 0.15 and 1, where the convergence was assured (S4 FigA). Across semisynthetic datasets, the hyperparameter λ that minimized $f_{obj}(\lambda)$ was greater than or equal to 0.5 (0.73 ± 0.19, mean ± s.d.; see S4 FigB)." (lines 311-322)

COMMENT 3-3.
What is the real-world application of the proposed *F*-MDS? Demonstrating a concrete application would make the importance of your method more convincing, particularly since it prioritizes preserving group differences even at the expense of some loss in the original distance structure.

RESPONSE 3-3.
The proposed method can be applied to settings in which low-dimensional visualizations fail to reflect the group differences detected by permutational statistical testing. Beyond the microbiome research originally proposed in the paper, we have now expanded the discussion of potential application areas to include metabolomics, neuroimaging, and pharmaceutical manufacturing by adding a new paragraph in the Discussion, which reads as follows:

> "… In microbiome research, for example, applying *F*-informed MDS to bacterial community datasets can allow users to interpret biologically meaningful patterns with higher confidence. It is also possible that the method could be employed in other domains where multivariate signals are processed based on distance metrics, such as metabolomics data clustering [70], network connectivity analysis with spatial neuroimaging [71], or spectral analysis for pharmaceutics quality control [72]." (lines 473-478)

COMMENT 3-4.
Line 184: "with all but one feature mean differently adjusted for each dataset" seems unclear. It appears that you intend to say that only one feature is set to have different means between the two groups, but the current wording implies that all features except one have different means. In addition, why did you choose to set only one differential feature? This seems too few given that there are 332 features in total.

RESPONSE 3-4.
We understand if the number of adjusted features in the semisynthetic datasets seems too few (e.g., one for binary datasets; five for ternary). Our goal in the simulation studies was to verify the proposed MDS using data that realistically captured microbiome characteristics as much as possible. Noting that SparseDOSSA's trained model with human stool microbiome data has shown a good fit (Ma et al., PLoS Comput. Biol., 2021), we chose to minimally perturb the model structure to realistically simulate the data.

In response, we have added a sentence in the main text discussing why the model adjustment was minimal (copied below). We again appreciate the reviewer's attention to detail.

> "In summary, the stool-trained SparseDOSSA model was adjusted for each data group to illustrate cases where metric MDS failed to reveal group-based patterns in a two-dimensional representation. The adjustments were designed to be minimal to capture the microbiome characteristics as realistically as possible." (lines 203-206)

COMMENT 3-5.
It is unclear which results (tables and figures) correspond to which datasets, as you introduce multiple semi-synthetic and real datasets in the "Dataset and Evaluation" section, but these datasets are not explicitly referenced in the Results section.

RESPONSE 3-5.
We apologize for the unclear description in the main text. For each figure and table caption in the Results section, we have now specified which dataset (i.e., binary/ternary semisynthetic or real microbiome) corresponds to each result. These captions are highlighted in pages 12, 13, 15, 17 and 19 of the marked-up copy.
