# Supplementary material for: Multidimensional scaling informed by F-statistic: Visualizing grouped microbiome data with inference
Source: PLoS Comput Biol. 2026 Apr 1;22(4):e1014102. doi: 10.1371/journal.pcbi.1014102 (PMC13108906; doi:10.1371/journal.pcbi.1014102)
Supplement: Attachment — Submitted filename: Response to Reviewers_Feb2026.pdf [file pcbi.1014102.s014.pdf]

Response to Comments by Referee #3

COMMMENT 3-1.
I do not understand why it is reasonable to order the F-values. Consider the extreme case in which the original F_z and F_x are perfectly negatively linearly correlated, which appears to be favored by your method since it yields a perfectly linear relationship. Is this the intended outcome?
Alternatively, consider this from another perspective. Since independent permutations can be applied to **Z** and **X**, F_z and F_x may be independent. Suppose there are two representations with the same raw stress. In this case, how does the confirmatory term help to reveal group differences and decide which representation is better?

RESPONSE 3-1.
We again appreciate the reviewer's feedback. The purpose of ordering *F*-statistics is to match their distributions, not to correlate every instance. We treat the *F*-statistics as distributions characterizing group separation capabilities. This ordering constructs the "Q-Q plot" required to predict the significance p_z in the reduced space from the original significance p_x, based on the Wasserstein distance at that epoch. This allows us to enforce that the inference in the reduced space Z remains consistent with the original space X, regardless of how individual permutation instances are paired.

Case 1 (Negative correlation): While ordering forces a monotonic relationship, this is an intentional consequence of the mapping described above. We are not matching specific permutation instances; we are mapping the probability of observing specific separation statistics. Therefore, the original pairing (or negative correlation) of raw instances is irrelevant to the goal of aligning statistical inference.

Case 2 (Tie-breaking): The confirmatory term resolves geometric ambiguities. If two representations have identical raw stress (same geometric shape), the confirmatory term favors the solution that yields a p_z closer to p_x. By minimizing the discrepancy in statistical significance, the algorithm selects the representation that best reflects the group separation confidence of the original data.

COMMENT 3-2.
The algorithm does not appear to converge. In Figure 2 of your response, p_z exhibits substantial and seemingly random fluctuations.

RESPONSE 3-2.
We apologize for the confusion regarding the term "convergence" in the main text. We acknowledge that the algorithm does not converge and instead oscillates. However, it does not contradict our main point, as we have empirically shown that an optimal solution consistently exists and its behavior has been extensively characterized. We demonstrated that by applying a region of validity $|p_z - p_x| < 0.05$, the final representation Z stabilizes without any fluctuations (S2 Fig B, copied below).

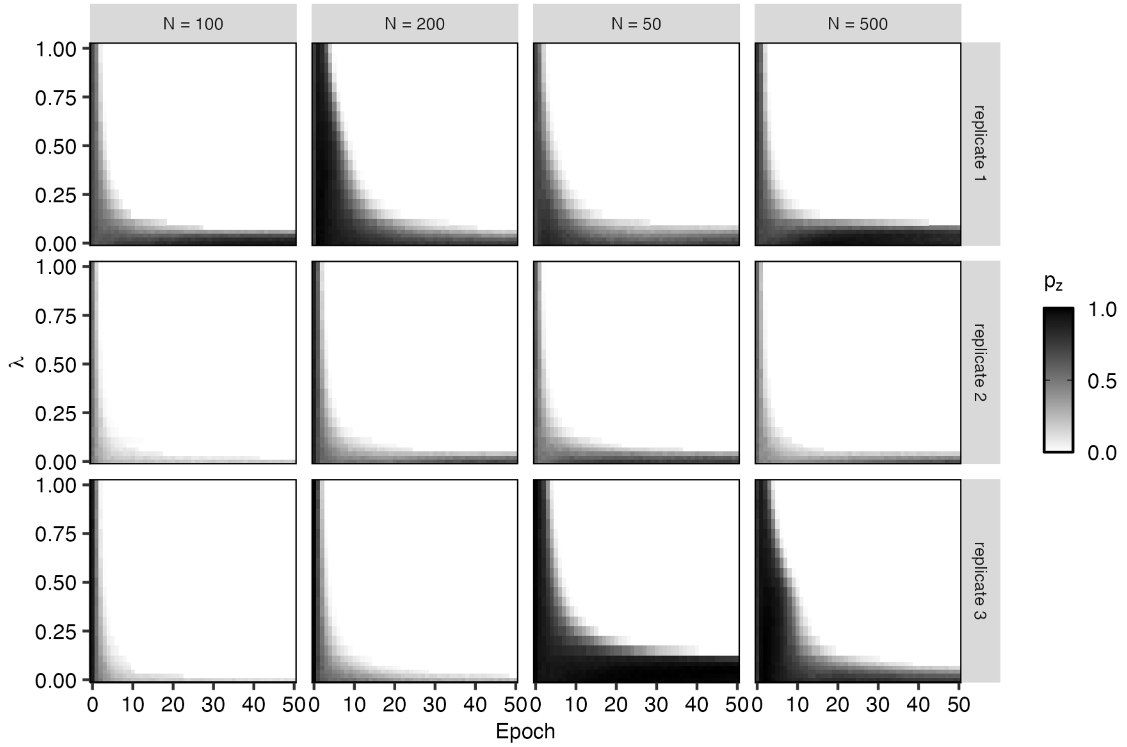

Copy of S2 Fig (B): Trajectories of *p*-values with the stopping rule ($|p_z - p_x| < 0.05$). Heatmaps show p_z values plotted against training epochs for hyperparameters $\lambda \in [0, 1]$. Triplicate datasets of varying sizes (*N* = 50, 100, 200, 500) were evaluated to demonstrate consistent stabilization across conditions.

We have revised the manuscript by replacing the word "convergence" to avoid ambiguity (highlighted in the marked-up version). Additionally, we clarified the necessity of the stopping criterion ($|p_z - p_x| < 0.05$) in the main text to ensure the algorithm terminates without unnecessary oscillation. The revised text reads:

> "… When training continued without a stopping rule, p_z-values oscillated between 0 and 1, with a higher oscillation rate at larger λ (S2 FigA). Therefore, we specified a bounded region, $| p_z - p_x | < 0.05$, as the stopping criterion for terminating the majorization algorithm (S2 Appendix)." (lines 270-271)
