# Supplementary material for: Multidimensional scaling informed by F-statistic: Visualizing grouped microbiome data with inference
Source: PLoS Comput Biol. 2026 Apr 1;22(4):e1014102. doi: 10.1371/journal.pcbi.1014102 (PMC13108906; doi:10.1371/journal.pcbi.1014102)
Supplement: Attachment — Submitted filename: PCOMPBIOL-D-25-00623_review.docx [file pcbi.1014102.s011.docx]

The authors propose a new MDS-based ordination method, F-informed MDS, which incorporates the F-statistic to guide dimensionality reduction in microbial ecology data. This method adds a supervised component to traditional ordination, which typically does not utilize group labels. While the approach is interesting and potentially useful, I have several concerns and questions:

1. The method relies on predefined group labels to compute the F-statistic. How would the method perform when such labels are unavailable or when there is no clear group separation in the data?

2. As noted earlier in the manuscript, microbiome data are typically compositional and often zero-inflated. However, it appears that the simulated data used to validate the method did not account for these features.

3. The simulations assume a diagonal covariance matrix, implying no correlation among microbial features. In reality, microbial taxa often exhibit complex interactions—both positive and negative correlations. This simplifying assumption may limit the generalizability of the method’s performance in real-world data.

4. Microbiome datasets are frequently high-dimensional. It is unclear how the proposed method performs in such scenarios. Are there any computational or numerical stability issues when the number of features exceeds the number of samples?

5. The manuscript explores the effect of different λ values through a grid search. Would it be possible to adopt a cross-validation framework to select λ in a more scientific approach?

6. How does F-informed MDS behave when there is no actual difference between the groups (i.e., under the null)? Does it still produce a stable embedding?
